# Exploiting Substrate Promiscuity of Ectoine Hydroxylase for Regio- and Stereoselective Modification of Homoectoine

**DOI:** 10.3389/fmicb.2019.02745

**Published:** 2019-11-27

**Authors:** Laura Czech, Sarah Wilcken, Oliver Czech, Uwe Linne, Jarryd Brauner, Sander H. J. Smits, Erwin A. Galinski, Erhard Bremer

**Affiliations:** ^1^Laboratory for Microbiology, Department of Biology, Philipps-Universität Marburg, Marburg, Germany; ^2^Department of Chemistry, Philipps-Universität Marburg, Marburg, Germany; ^3^Institute of Microbiology and Biotechnology, Rheinische Friedrich-Wilhelms-Universität, Bonn, Germany; ^4^Institute of Biochemistry, Heinrich-Heine Universität Düsseldorf, Düsseldorf, Germany; ^5^Center for Structural Studies, Heinrich-Heine Universität Düsseldorf, Düsseldorf, Germany; ^6^SYNMIKRO Research Center, Philipps-Universität Marburg, Marburg, Germany

**Keywords:** compatible solutes, ectoine, homoectoine, ectoine hydroxylase, biocatalysis, whole cell biotransformation, chemical biology

## Abstract

Extant enzymes are not only highly efficient biocatalysts for a single, or a group of chemically closely related substrates but often have retained, as a mark of their evolutionary history, a certain degree of substrate ambiguity. We have exploited the substrate ambiguity of the ectoine hydroxylase (EctD), a member of the non-heme Fe(II)-containing and 2-oxoglutarate-dependent dioxygenase superfamily, for such a task. Naturally, the EctD enzyme performs a precise regio- and stereoselective hydroxylation of the ubiquitous stress protectant and chemical chaperone ectoine (possessing a six-membered pyrimidine ring structure) to yield *trans*-5-hydroxyectoine. Using a synthetic ectoine derivative, homoectoine, which possesses an expanded seven-membered diazepine ring structure, we were able to selectively generate, both *in vitro* and *in vivo*, *trans*-5-hydroxyhomoectoine. For this transformation, we specifically used the EctD enzyme from *Pseudomonas stutzeri* in a whole cell biocatalyst approach, as this enzyme exhibits high catalytic efficiency not only for its natural substrate ectoine but also for homoectoine. Molecular docking approaches with the crystal structure of the *Sphingopyxis alaskensis* EctD protein predicted the formation of *trans*-5-hydroxyhomoectoine, a stereochemical configuration that we experimentally verified by nuclear-magnetic resonance spectroscopy. An *Escherichia coli* cell factory expressing the *P. stutzeri ectD* gene from a synthetic promoter imported homoectoine *via* the ProU and ProP compatible solute transporters, hydroxylated it, and secreted the formed *trans*-5-hydroxyhomoectoine, independent from all currently known mechanosensitive channels, into the growth medium from which it could be purified by high-pressure liquid chromatography.

## Introduction

It is generally assumed that primordial cells had only a restricted number of proteins with different folds and that the enzymes present in these cells exhibited a broad substrate specificity (Jensen, 1976; Khersonsky and Tawfik, 2010; Michael, 2017). This substrate ambiguity (Jensen, 1976) provided fertile ground for evolution to shape the substrate profiles of enzymes in extant microbial cells toward a higher specificity and catalytic efficiency (Jensen, 1976; Khersonsky and Tawfik, 2010; Pandya et al., 2014; Michael, 2017; Newton et al., 2018). However, it is increasingly recognized that many of the extant enzymes have retained, at least in part, their original substrate ambiguity, thereby creating a metabolic profile of cells that is fashionably addressed as “underground metabolism” (D’Ari and Casadesus, 1998). While imposing a metabolic burden onto the cell, enzyme promiscuity allows the selection of novel metabolic traits when new substrates become available, or relieve constrains in metabolism when bottlenecks arise. Underground metabolism, combined with selective pressures on growth and survival, can thus aid the adaptation of microorganisms to new ecological niches (Barrick and Lenski, 2013; Pandya et al., 2014; Michael, 2017; Newton et al., 2018).

Enzyme promiscuity can also be harnessed in biotechnological applications, long-term evolution experiments for pathway development, shaping of metabolic networks for the production of useful compounds, and the engineering of enzymes with tailor-made functions (Nobeli et al., 2009; Michael, 2017; Guzman et al., 2019; Rosenberg and Commichau, 2019). The substrate ambiguity of enzymes can also be “hijacked” for the biotransformation and production of man-made compounds (D’Ari and Casadesus, 1998). In this study, we have exploited the biochemical properties of the ectoine hydroxylase (EctD) for such a task. Naturally, this enzyme catalyzes the synthesis of the stress protectant and chemical chaperone 5-hydroxyectoine from its precursor ectoine (Bursy et al., 2007; Höppner et al., 2014).

**FIGURE 1:**
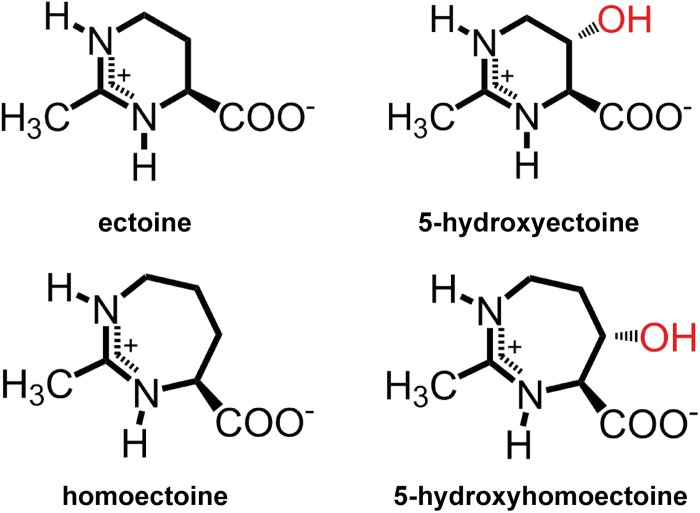
Chemical structures of the compatible solutes ectoine, 5-hydroxyectoine, homoectoine, and 5-hydroxyhomoectoine.

Ectoine [(*S*)-2-methyl-1,4,5,6-tetrahydropyrimidine-4- carboxylic acid] (Galinski et al., 1985) and its derivative 5-hydroxyectoine [(4*S*,5*S*)-5-hydroxy-2-methyl-1,4,5,6-tetrahydro -pyrimidine-4-carboxylic acid] (Inbar and Lapidot, 1988) (Figure 1) are prominent members of the so-called compatible solutes, a special class of highly water-soluble organic osmolytes that are compliant with cellular biochemistry and physiology (da Costa et al., 1998; Bolen and Baskakov, 2001; Roberts, 2004). Microorganisms use them widely as osmostress protectants (Galinski and Trüper, 1994; Kempf and Bremer, 1998; Roesser and Müller, 2001; Wood et al., 2001; Gunde-Cimerman et al., 2018). When faced with high osmolarity surroundings, many bacteria accumulate compatible solutes to counteract the outflow of water from the cell, and thereby prevent dehydration of the cytoplasm, increase in molecular crowding, and drop in vital turgor to physiologically unsustainable values (Wood, 2011; van den Berg et al., 2017; Bremer and Krämer, 2019). The amassing of these osmostress protectants can occur either through synthesis or import (Kempf and Bremer, 1998; Bremer and Krämer, 2000), but their uptake is generally preferred for energetic reasons (Oren, 1999).

In addition to their pure osmostress adaptive role, compatible solutes protect the functionality of proteins, macromolecular complexes, membranes, and even entire cells, have broad protein anti-aggregating properties, and can influence DNA structure (Lippert and Galinski, 1992; Barth et al., 2000; Bourot et al., 2000; Ignatova and Gierasch, 2006; Harishchandra et al., 2010; Meyer et al., 2017; Stadmiller et al., 2017). These biological function-preserving attributes have led to their description as chemical chaperones (Diamant et al., 2001; Chattopadhyay et al., 2004). The beneficial effects of compatible solutes on stability and functionality of proteins are generally explained in the framework of the preferential exclusion model (Arakawa and Timasheff, 1985). The unfavorable interactions of compatible solutes with the protein backbone (Liu and Bolen, 1995) and the ensuing uneven distributions of these solutes in the surrounding water, in conjunction with the modification of the solvation properties of the solvent, lead, for thermodynamic reasons, to well-hydrated and well-folded proteins, a process that has been coined the osmophobic effect (Bolen and Baskakov, 2001). Hence, compatible solutes act against the unfolded state of proteins under intracellular unfavorable osmotic and ionic conditions (Bolen and Baskakov, 2001; Ignatova and Gierasch, 2006; Street et al., 2006; Capp et al., 2009; Zaccai et al., 2016; Stadmiller et al., 2017). In this respect, the function-preserving and anti-inflammatory attributes of ectoines have attracted particular attention, and ectoines are therefore increasingly exploited for various practical applications (Lentzen and Schwarz, 2006; Graf et al., 2008; Pastor et al., 2010; Kunte et al., 2014).

Ectoine is synthesized from the central microbial metabolite L-aspartate-β-semialdehyde (Lo et al., 2009; Stöveken et al., 2011) in a three-step biosynthetic route involving L-2,4-diaminobutyrate (DAB) transaminase (EctB), L-2,4-diaminobutyrate acetyltransferase (EctA), and ectoine synthase (EctC) (Peters et al., 1990; Ono et al., 1999). In a substantial sub-group of ectoine-producing bacteria (Czech et al., 2018a), ectoine is chemically modified by the ectoine hydroxylase (EctD) to *trans*-5-hydroxyectoine (Prabhu et al., 2004; Garcia-Estepa et al., 2006; Bursy et al., 2007). From a chemical point of view, the regio- and stereoselective modification of ectoine by a hydroxyl group (Inbar and Lapidot, 1988; Bursy et al., 2007) seems to be a rather minor alteration. However, it can have profound consequences with respect to the influence of 5-hydroxyectoine on water structure and its solubility at different temperatures; it often also results in superior function-preserving attributes compared to ectoine (Held et al., 2010; Smiatek et al., 2012; Hahn et al., 2015; Czech et al., 2018a). Examples are superior protective effects against desiccation, the ability to form glasses, and the stabilization of and influence on DNA, proteins, and lipid layers (Lippert and Galinski, 1992; Borges et al., 2002; Manzanera et al., 2004; Harishchandra et al., 2010, 2011; Tanne et al., 2014).

Among all compatible solutes considered for practical uses (Jorge et al., 2016), ectoines have found the widest applications, and numerous products are already commercially available (Graf et al., 2008; Pastor et al., 2010; Kunte et al., 2014). Ectoines have found uses in cosmetics, skin care, and the stabilization of cells and recombinant proteins; their applications in medicine are actively pursued. Ectoines are currently provided through an industrial-scale biotechnological production process that is able to deliver ectoine on the scale of tons by using *Halomonas elongata* (Schwibbert et al., 2011) as either natural or engineered cell factory (Lentzen and Schwarz, 2006; Pastor et al., 2010; Kunte et al., 2014). Ectoines are commercially high-value products, inspiring not only the development of recombinant microbial cell factories for their production (Giesselmann et al., 2019), but also the design and chemical synthesis of ectoine derivatives. These derivatives possess either reduced or expanded sizes of the six-membered ectoine ring structure (Figure 1), or were modified with a lipid anchor (lauryl-ectoine) to alter the cellular targeting of the otherwise highly water soluble (up to 7 M) ectoines in eukaryotic cells (Schnoor et al., 2004; Held et al., 2010; Witt et al., 2011; Wedeking et al., 2014).

The synthetic ectoine derivative homoectoine [4,5,6,7-tetrahydro-2-methyl-1H-(1,3)-diazepine-4-carboxylic acid (homoectoine)] (Figure 1), the focus of this study, serves as an osmostress protectant for *Escherichia coli*, and functions as a potent PCR enhancer (Nagata, 2001; Schnoor et al., 2004; Shi and Jarvis, 2006). Notably, it also provides protection against colitis in mice and reduces intestinal inflammation, thereby raising the prospect of medical and other types of practical applications for homoectoine (Castro-Ochoa et al., 2019; Farre and Vicario, 2019). Compared with ectoine, a six-membered pyrimidine ring, the synthetic homoectoine molecule possesses a seven-membered diazepine ring (Figure 1).

**FIGURE 2:**
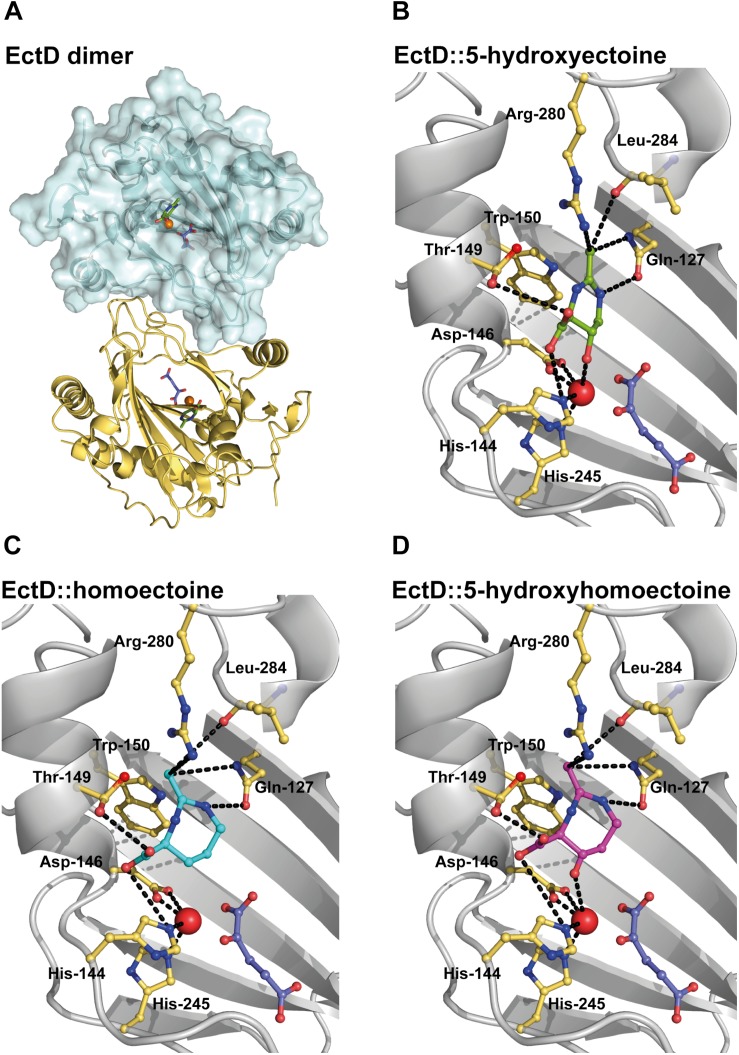
Modeling and docking of different substrates into the crystal structure of the ectoine hydroxylase EctD from *S. alaskensis.* **(A)** The overall fold of the (*Sa*)EctD (PDB accession code: 4Q5O) is shown as a dimer with one monomer presented as the surface structure of the protein and the other monomer in cartoon representation. Each monomer harbors the product, 5-hydroxyectoine (green) of the (*Sa*)EctD-catalyzed enzyme reaction, the co-substrate 2-oxoglutarate (light blue), and the catalytically important ion metal (red ball). Zoom into the active site of (*Sa*)EctD with **(B)** the bound natural reaction product 5-hydroxyectoine (green), **(C)** the modeled synthetic substrate homoectoine (blue), and **(D)** the modeled synthetic reaction product 5-hydroxyhomoectoine (pink). The amino acids involved in substrate binding are shown as yellow sticks and possible interactions are indicated by black dotted lines.

The fact that the hydroxylation of ectoine often attains superior function-preserving attributes kindled interests to hydroxylate synthetic ectoine derivatives as well (Galinski et al., 2009, 2015). This brings the properties of the ectoine hydroxylase EctD into focus (Prabhu et al., 2004; Garcia-Estepa et al., 2006; Bursy et al., 2007). Biochemical and crystallographic analysis revealed that EctD is a member of the non-heme Fe(II)-containing and 2-oxoglutarate-dependent dioxygenase superfamily (Bursy et al., 2007; Reuter et al., 2010; Höppner et al., 2014; Widderich et al., 2014a, 2016). Like other dioxygenases (Aik et al., 2012; Hangasky et al., 2013; Herr and Hausinger, 2018), EctD is a cupin and introduces a hydroxyl group to an inactive carbon of ectoine through a highly reactive ferryl species that is generated from the Fe(II) catalyst, the α-ketoglutarate co-substrate, and molecular O_2_ (Widderich et al., 2014b). EctD forms a dimer in solution and in the crystal (Figure 2A). All residues important for substrate/co-substrate binding and enzyme catalysis protrude into the lumen of the cupin barrel that also contains the catalytically critical Fe(II) atom (Höppner et al., 2014; Widderich et al., 2014b) (Figure 2B). Hence, the large barrel-like structure of the EctD monomer might be conducive to accommodating and accurately positioning compounds structurally and chemically related to ectoines and proline (Galinski et al., 2009, 2015; Hara et al., 2019).

The promiscuous activity of biosynthetic enzymes can be exploited for the resources-preserving production of valuable synthetic compounds by microorganisms. With a focus on the ectoine hydroxylase, we harnessed the substrate promiscuity of this enzyme for a regio- and stereoselective hydroxylation of the synthetic ectoine derivative homoectoine in a recombinant *E. coli* strain that secretes the newly formed *trans-*5-hydroxyhomoectoine into the growth medium in a process independent of all currently known mechanosensitive channels.

## Materials and Methods

### Chemicals

Ectoine was a kind gift from bitop AG (Witten, Germany). 5-Hydroxyectoine and glycine betaine were purchased from Merck KGaA (Darmstadt, Germany). Homoectoine was synthesized according to previously reported procedures (Koichi et al., 1991; Schnoor et al., 2004). Dithiothreitol (DTT) was purchased from AppliChem (Darmstadt, Germany) and phenylmethylsulfonyl fluoride (PMSF) from Roche (Basel, Switzerland). Desthiobiotin, Strep-TactinSuperflow chromatography material for the purification of proteins fused to a *Strep*-tag II affinity peptide and anhydrotetracycline hydrochloride (AHT) for the induction of the transcriptional activity of the TetR-regulated *tet* promoter present on the *ectD* expression plasmids pMP40 (*ectD* from *Sphingopyxis alaskensis*) and pMP41 (*ectD* from *Pseudomonas stutzeri*) (Widderich et al., 2014a) were purchased from IBA GmbH (Göttingen, Germany). All other chemicals were purchased from Karl Roth GmbH (Karlsruhe, Germany), Merck KGaA (Darmstadt, Germany), and Sigma-Aldrich (Steinheim, Germany).

### Bacterial Strains, Construction of *E. coli* Mutants and Plasmids

Standard genetic methods such as phage P1*vir*-mediated transduction were performed as described previously (Miller, 1972). For the construction of *E. coli* strain LC11 and LC12, P1*vir* phage lysates were prepared on cells of strains MKH17 [Δ(*proU:spc*)608] and JW4072-1 [Δ(*proP:kan*)737], respectively (Haardt et al., 1995; Baba et al., 2006). These were then used to transduce the *E. coli* K-12 wild-type strain MG1655 (Blattner et al., 1997) (Supplementary Table S1) by selecting for spectinomycin-resistant or kanamycin-resistant colonies on LB agar plates containing 100 μg ml^–1^ or 50 μg ml^–1^ of the antibiotic, respectively. Representative colonies were picked and purified by re-streaking several times on spectinomycin- or kanamycin-containing LB agar plates. The resulting strains were LC11 [Δ(*proU:spc*)608 *proP*^+^] and LC12 [Δ(*proP:kan*)737 *proU*^+^], respectively. Strain LC11 [Δ(*proU:spc*)608] was subsequently transduced with a P1*vir* phage lysate prepared on strain JW4072-1 [Δ(*proP:kan*)737] (Kitagawa et al., 2005) to obtain a double-gene deletion strain defective in the osmoprotectant uptake systems ProP and ProU (Lucht and Bremer, 1994; Haardt et al., 1995); the resulting strain was named LC14 [Δ(*proU:spc*)608 Δ(*proP:kan*)737] (Supplementary Table S1). To construct the *E. coli* K-12 strain LC15 that is deficient in the synthesis of the osmostress protectant trehalose, a P1*vir* phage lysate was prepared on cells of strain FF4169 [(*otsA*:Tn*10*)1] (Giaever et al., 1988) and was then used to transduce the defect in trehalose synthesis into the *otsBA*^+^ *E. coli* strain MG1655 by selecting for tetracycline-resistant colonies on LB agar plates containing 15 μg ml^–1^ of the antibiotic. To test the contribution of mechanosensitive channels in the release of the hydroxylated ectoine derivatives, a pair of isogenic *E. coli* strains Frag1 (parent) and a mutant (MJF641), which lacks all currently known mechanosensitive channels, was used (Edwards et al., 2012). All strains used in this study and their genotypes are listed in Supplementary Table S1.

Plasmids carrying the *ectD* gene from *P. stutzeri* A1501 (accession number: ABP77885.1) or from *S. alaskensis* (accession number: WP_011543221.1), pMP41 and pMP40, respectively, were used to overproduce the ectoine hydroxylases in the *E. coli* B strain BL21(DE3) (Studier et al., 1990) for their subsequent biochemical characterization (Widderich et al., 2014a). All plasmids used in this study are listed in Supplementary Table S2.

### Media and Growth Conditions for Osmostress Protection Assays

The *E. coli* K-12 strain MG1655 and its mutant derivatives LC11, LC12, LC14, and LC15 (Supplementary Table S1) were routinely maintained on LB agar plates and incubated at 37°C. For osmostress protection growth assays (Haardt et al., 1995), the strains were inoculated in 5 ml LB medium. After aerobic incubation for 5 h at 37°C, 100 μl culture was transferred to 10 ml MMA containing either no additional NaCl or 0.3 M NaCl with the aim to pre-adapt the *E. coli* cells to increased salinity. These cultures were then grown aerobically over night at 37°C and used to inoculate the main cultures (20 ml in 100-ml Erlenmeyer flasks) in a chemically defined medium (MMA) (Miller, 1972) supplemented with 0.5% glucose, 1 mg l^–1^ thiamine, and 1 mM MgSO_4_ to an OD_578_ of 0.1. These cultures contained either no NaCl (control) or 0.8 M NaCl (osmotic stress). Compatible solutes (ectoine, 5-hydroxyectoine, homoectoine, hydroxyhomoectoine, and glycine betaine) were added to high-salinity growth media to a final concentration of 1 mM. The OD_578_ values of the various cultures were measured after 10, 24, 30, 36, and 48 h in technical duplicates. For the visualization of the growth of cultures, the pre-cultures were grown under the same conditions as mentioned above and the main cultures were incubated in a well-plate reader (Epoch2, BioTek) for 72 h at 37°C with constant double-orbital shaking. The culture volume per well was 500 μl with an end concentration of compatible solutes of 1 mM. The OD_578_ was measured every hour. Each growth experiment was performed in duplicate and representative growth curves are presented in this manuscript.

### *In vivo* Biotransformation of Ectoine Into 5-Hydroxyectoine and Homoectoine Into 5-Hydroxyhomoectoines

Biotransformation assays for various ectoines were conducted as previously described (Czech et al., 2016) in an *E. coli* MG1655 strain background. Strain LC15 (MG1655 *otsA*:Tn*10*) (Supplementary Table S1) harboring the *ectD*^+^ plasmid pMP41 (or pASK-IBA3 as the negative control) (Supplementary Table S2) was inoculated in 5 ml LB medium containing 100 μg ml^–1^ ampicillin and grown aerobically at 37°C for 5 h; 100 μl of this pre-culture was used to inoculate 10 ml of MMA without NaCl which was subsequently incubated at 37°C over night. Main cultures were grown in baffled flasks (100 ml) containing 10 ml of MMA with 0.4 M NaCl and various concentrations of ectoine or homoectoine. Cells were grown to an OD_578_ of 0.5 when 0.2 mg l^–1^ of the inducer (AHT) of the TetR repressor was added to trigger enhanced activity of the plasmid-based *tet* promoter driving *ectD* transcription; the cultures were further grown for 24 h at 37°C. After this time, 2 ml per culture was harvested in duplicates, and supernatants were stored at −20°C until further use. Extracellular concentrations of ectoine and its derivatives in the supernatant of the cultures were analyzed by high-performance liquid chromatography (HPLC). In order to address the role of mechanosensitive channels (Booth, 2014; Cox et al., 2018) in the release/excretion of hydroxylated derivatives of ectoine or homoectoine, the same types of experiments were conducted with the isogenic strains Frag1 and MJF641 (Supplementary Table S1). Strain MJF641 lacks all currently known mechanosensitive channels (Edwards et al., 2012).

### HPLC-Based Analysis of Ectoines

For the HPLC-based quantification of ectoines, an Agilent 1260 Infinity LC system (Agilent, Waldbronn, Germany) was employed. Prior to loading of the samples into the HPLC system, they were diluted 1:2 with acetonitrile and measured as reported previously (Kuhlmann and Bremer, 2002; Czech et al., 2016). For separation and detection of various ectoines, a GROM-SIL Amino-100 PR column (3 μm) (obtained from Dr. Maisch HPLC GmbH, Ammerbuch-Entringen, Germany) with an attached 1260 Infinity Diode Array Detector system was used. Absorbance of the compatible solutes was measured at a wavelength of 210 nm. Commercially available samples of ectoine and 5-hydroxyectoine were used to prepare standard solutions. Separation of homoectoine and hydroxyhomoectoine was achieved by using a gradient of water and acetonitrile as the mobile phase. The portion of water being mixed with acetonitrile during each measurement was increased from 5 up to 30% within 17 min. Chromatograms of the HPLC runs were analyzed with the OpenLAB software suite (Agilent) and ectoine concentrations in individual samples were determined from measured reference standards and by using the programs Excel (Microsoft) and GraphPad Prism (GraphPad Software, La Jolla, CA, United States^1^).

### Detection of 5-Hydroxyhomoectoine in the Supernatant by Mass Spectrometry

In order to confirm the hydroxylation of homoectoine, the supernatants of two biologically independent *E. coli* cell cultures producing ectoine hydroxylases were analyzed by HPLC and subsequently by mass spectrometry. The HPLC conditions were the same as described above. As controls, the supernatants of a cell factory harboring the empty vector pASK-IBA3 (IBA GmbH, Göttingen) and of a culture without compatible solutes were measured. HR-ESI mass spectra were acquired with a LTQ-FT Ultra mass spectrometer (Thermo Fisher Scientific). The resolution was set to 100.000. Data were evaluated using Xcalibur (Thermo Fisher Scientific).

### HPLC-Based Preparation of 5-Hydroxyhomoectoine

The supernatant of an *ectD*^+^ *E. coli* culture producing 5-hydroxyhomoectoine was used to prepare this compound. For this purpose, the complete supernatant of the culture (10 ml) that had received 5 mM homoectoine and had been grown for 24 h was step-wise loaded on a GROM-SIL Amino-100 PR column (3 μm) and the separation of 5-hydroxyhomoectoine was monitored at 210 nm (Kuhlmann and Bremer, 2002; Czech et al., 2016). The elution peak of 5-hydroxyhomoectoine was automatically collected using a fraction collector (1100 series, Agilent). Because an analytical column was used, only 25 μl of the supernatant could be analyzed in a single run due to observed overloading effects. Combined fractions containing pure 5-hydroxyhomoectoine were subsequently lyophilized and stored at −20°C. The 5-hydroxyhomoectoine purified from the supernatant of two 10 ml cultures (14 mg dry weight) was dissolved in 780 μl dH_2_O resulting in a concentration of 100 mM and was used as a HPLC standard solution, and for osmostress protection assays. Due to the observed instability of 5-hydroxyhomoectoine at neutral pH (Supplementary Figure S1), the very first measured standard curve of 5-hydroxyhomoectoine was subsequently used to achieve the most reliable quantification of 5-hydroxyhomoectoine in HPLC assays. A second round of 5-hydroxyectoine purification from the supernatants of two *ectD* expressing cultures yielded again approximately 14 mg of 5-hydroxyhomoectoine; they were dissolved in 700 μl water and this solution was employed for NMR analysis.

### Stereochemical Analysis of Hydroxyhomoectoine by NMR

The purified 5-hydroxyhomoectoine was employed to elucidate the stereochemical configuration of hydroxyhomoectoine, in particular to reveal the carbon atom at which the hydroxylation takes place and with which selectivity. Broad-band-decoupled ^1^H NMR spectra and 2d-NMR spectra were recorded at 300 K on a Bruker AV III HD 500 MHz spectrometer in deuterium oxide (99.9% D) containing 3-(trimethylsilyl)propionic-2,2,3,3-d4 acid sodium salt (internal standard) (Sigma-Aldrich Chemie GmbH, München, Germany) at 500.13 MHz or on a Bruker AV II 600 MHz in H_2_O using solvent suppression excitation sculpting with gradients at 600.13 MHz. ^13^C NMR spectra were recorded on a Bruker AV II 300 at 75.49 MHz. Chemical shifts are reported in ppm relative to the solvent-residue signal (^1^H spectra) or to the internal standard (^13^C spectra), respectively. Multiplicities are given as singlet (s), doublet (d), triplet (t), quartet (q), quintet (quin), multiplet (m), and broad (b) where applicable. Spectra were processed with Bruker TopSpin^®^ software 4.0.

### Density Functional Theory Calculations

Density functional theory (DFT) calculations were performed with Gaussian 2016 Rev. A.03^2^ employing the Lee Yang Parr hybrid functional B3LYP (Becke, 1993) and the 6-311 + G^∗∗^ (Wachters, 1970) split-valence basis set with added polarization and diffuse functions. The RMS force criterion was set to 10^–5^. All generated minima were verified to have zero imaginary frequency modes *via* analytic computation of the Hesse matrix. The computed coordinates of the (4*S*,5*S*)-isomer can be found in the Supplementary Material.

### Overproduction and Purification of Recombinant EctD Enzymes

For overproduction of the recombinant EctD enzymes, the *E. coli* strain BL21 (DE3) (Studier et al., 1990) harboring either the plasmid pMP40 (*ectD* gene from *S. alaskensis*) or pMP41 (*ectD* gene from *P. stutzeri* A1501) was used (Widderich et al., 2014a). In these plasmids, expression of the *ectD* genes is under the control of the *tet* promoter which responses in its transcriptional activity to the TetR repressor. Cells harboring one of the plasmids were grown in modified MMA (0.5% glucose, 1 mg ml^–1^ thiamine, 1 mM MgSO_4_, 0.5% casamino acids) to an OD_578_ of 0.5. AHT was then added to the cultures in a final concentration of 0.2 mg l^–1^ to induce *ectD* gene expression from the TetR-repressed *tet* promoter, and the cells were then further incubated for 2.5 h. The cultures were harvested by centrifugation (4°C, 5063 × g, 20 min) and re-suspended in 20 mM TES (pH 7.5) containing 100 mM KCl; the cells were subsequently pelleted by centrifugation for 10 min. The pelleted cells were stored at −20°C until further use. To purify the EctD-*Strep*-tag II recombinant proteins by affinity chromatography, the pellets of the overproducing cells were re-suspended in 5 ml 20 mM TES (pH 7.5), containing 100 mM KCl and protease inhibitors (2 mM DTT, 0.5 mM PMSF, 0.5 mM benzamidine). The cells were disrupted by passing them three times through a French Press at 1000 psi; the cell lysate was subsequently centrifuged for 1 h and at 21.000 × g at 4°C. EctD-*Strep*-tag II recombinant proteins were purified *via* a Strep-Tactin Superflow column according to a standard protocol (IBA GmbH, Göttingen). Elution of the recombinant EctD-*Strep*-tag II proteins was achieved by adding buffer [20 mM TES (pH 7.5), containing 100 mM KCl] containing desthiobiotin (2.5 mM) and no protease inhibitors. Enzyme fractions were stored at −80°C or directly used in EctD enzyme activity assays.

### EctD Enzyme Activity Assays

In order to characterize the conversion of ectoine into 5-hydroxyectoine and homoectoine into 5-hydroxyhomoectoine in a quantitative fashion, purified ectoine hydroxylases were used in enzyme activity assays. The assays had a volume of 30 μl and after optimization of the previously used conditions (Höppner et al., 2014; Widderich et al., 2014a), the following conditions were chosen: the reaction mixture contained 100 mM TES (pH 7.5), 1 mM FeSO_4_, 10 mM, 2-oxo-glutarate, 100 mM KCl, and various amounts of ectoine or homoectoine as substrate. The FeSO_4_ solution (dissolved in dH_2_O) was always prepared fresh. In case of enzyme assays conducted with the *S. alaskensis* (*Sa*) EctD protein, 1300 U catalase from bovine liver was added to the reaction assay. Before addition of the EctD enzyme, the reaction mixture was pre-incubated for 10 min at an optimal assay temperature of 35°C (for EctD from *P. stutzeri* A1501) or at 15°C (for EctD from *S. alaskensis*). The enzyme assays were started by adding 1.47 μM of the recombinant ectoine hydroxylase to the pre-mixed assay solution. The enzyme assays were stopped after 5 min by the addition of acetonitrile in a 1:2 ratio. Denatured proteins were removed by centrifugation at 13.000 × *g* for 10 min at room temperature. Ectoines were detected by HPLC analysis as described (Kuhlmann and Bremer, 2002; Czech et al., 2018b). The kinetic parameters were calculated and fitted according to Michaelis Menten kinetics using GraphPad Prism version 5 for MacOsX (GraphPad Software, La Jolla, CA, United States^1^).

### Bioinformatic Tools for Docking Simulations

The crystal structure of the EctD protein from *S. alaskensis* in complex with 5-hydroxyectoine (PDB accession code: 4Q5O) (Höppner et al., 2014) was used to dock homoectoine, hydroxyhomoectoine, and hydroxyproline into the active site of the enzyme. The structures of homoectoine, hydroxyhomoectoine, and hydroxyproline were built using the program Phenix (Afonine et al., 2012). With help of the program COOT (Emsley and Cowtan, 2004), the ligands were placed at five different positions in close proximity to the EctD protein structure. To circumvent any bias during the modeling process, the positions of the various ligands were chosen to be either within the active site (two positions) or at the surroundings of the EctD protein (three positions). These positions, together with the monomeric EctD structure in complex with Fe(II) (Höppner et al., 2014), were subjected to the automated software AUTODOCK using standard settings (Trott and Olson, 2010). The results of every docking run were manually inspected. The result showed in every case the binding of homoectoine at the same site as observed for 5-hydroxyectoine in the EctD crystal structure (Höppner et al., 2014). Subsequently, by changing the orientation of the homoectoine molecule within the EctD active site, a second round of docking was performed. This resulted in a stable conformation of the homoectoine ligand within the EctD active site in close proximity to the catalytically crucial iron (Höppner et al., 2014). The same procedure was performed using hydroxyhomoectoine and hydroxyproline. In all cases, the final result was manually inspected using the programs Pymol (Delano, 2002)^3^ and COOT (Emsley and Cowtan, 2004).

The volume of the cavity in the active site of (*Sa*)EctD (PDB accession code: 4MHR) (Höppner et al., 2014) was calculated using the program CastP (Tian et al., 2018) using a monomer of the dimeric EctD protein (Höppner et al., 2014) as the input crystal structure. To verify that the CastP program found the correct substrate-binding pocket of EctD, the calculated pocket was overlaid with the actual crystal structure of the EctD protein in complex with 5-hydroxyectoine (PDB accession code: 4Q5O). The same procedure was used with the (*Sa*)EctD structure to obtain models of EctD in complex with either homoectoine or 5-hydroxyhomoectoine.

### Protein Alignments and Figure Preparation

The protein sequence alignment of the EctD proteins from *S. alaskensis* (WP_011543221.1), *Acidiphilum cryptum* (WP_012040480.1), *Paenibacillus lautus* (WP_015737572.1), *H. elongata* (WP_013333764.1), *Streptomyces coelicolor* (NP_626134.1), *P. stutzeri* (WP_011911424.1), *Halobacillus halophilus* (WP_014643639.1), *Nitrosopumilus maritimus* (WP_ 012215726.1), *Chromohalobacter salexigens* (WP_011505850.1; WP_011508293.1), *Alkalilimnicola ehrlichii* (WP_011628142.1), *Streptomyces chrysomallus* (WP_030590139.1), and *Virgibacillus salexigens* (AAY29689.1) was performed with SnapGene^®^ software (GSL Biotech^4^).

All figures were prepared using either GraphPad Prism (GraphPad Software, La Jolla, CA, United States^1^), Adobe Illustrator^5^, or Pymol^3^ (Delano, 2002).

## Results

### Homoectoine Is an Osmostress Protectant for the *E. coli* Strain MG1655

**FIGURE 3:**
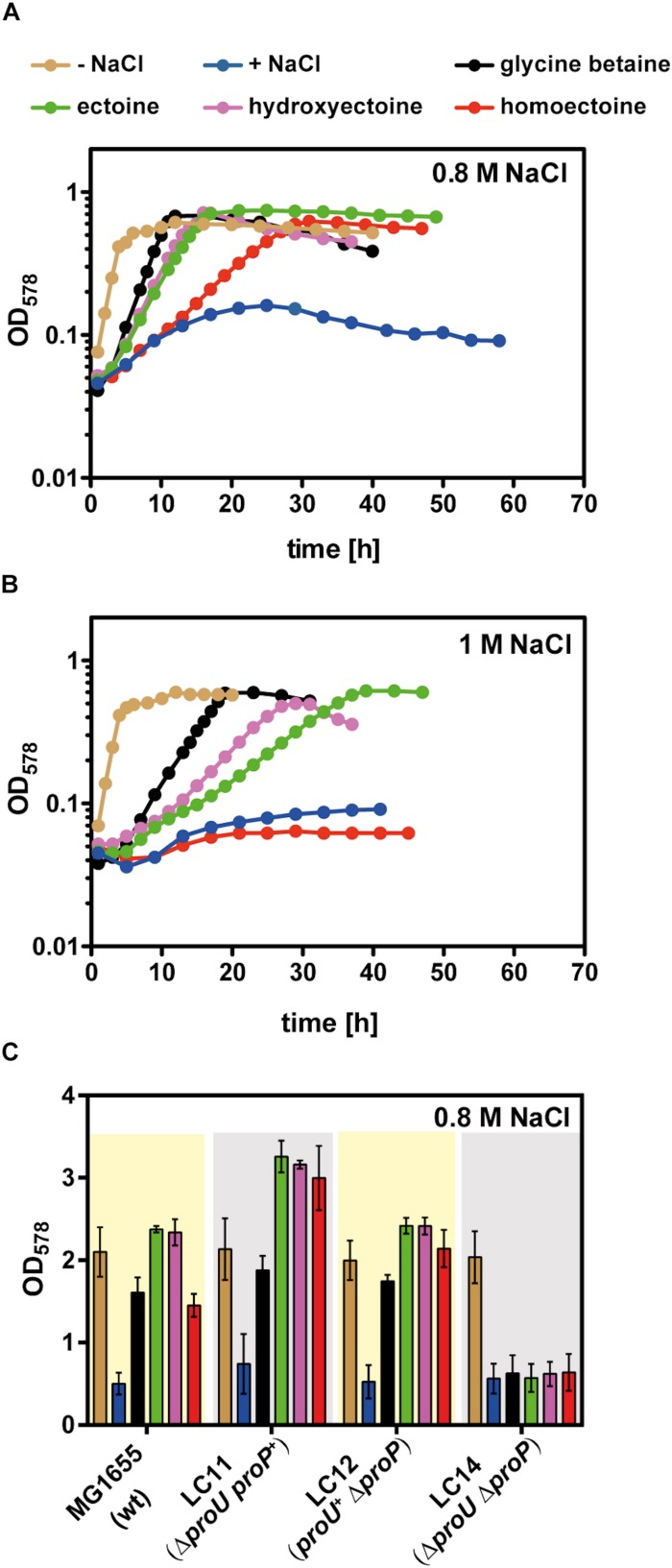
Osmostress protection assay with glycine betaine, ectoine, 5-hydroxyectoine, and homoectoine. Growth of the *E. coli* strain MG1655 (*proU*^+^ *proP*^+^) in MMA without NaCl and in MMA containing **(A)** 0.8 M NaCl or **(B)** 1 M NaCl and 1 mM of the indicated osmostress protectants; growth of the cultures was monitored for 60 h. The OD_578_ of the cultures was measured hourly in a plate reader (Epoch2, BioTek). Each osmoprotection assay was conducted with two independently grown cultures. A representative set of growth curves is shown. **(C)** The *E. coli* strains MG1655 (*proU*^+^ *proP*^+^), LC11 (*proU*^–^ *proP*^+^), LC12 (*proU*^+^ *proP*^–^), and LC14 (*proU*^–^ *proP*^–^) were grown either in MMA or MMA containing 0.8 M NaCl in the absence or presence of the osmostress protectants (1 mM) in shake flasks. The final growth yield was determined by measuring the OD_578_ of the cultures after 30 h. The bars shown are the means and standard deviations of three independently grown cultures.

*Escherichia coli* possesses two osmotically regulated uptake systems for a variety of compatible solutes: the proton-solute symporter ProP, a member of the major facilitator superfamily (MFS) and the ABC-type (ATP-binding cassette) transporter ProU (Lucht and Bremer, 1994; Wood et al., 2001). Both transporters can also serve as uptake systems for ectoine and 5-hydroxyectoine (Jebbar et al., 1992; Czech et al., 2016; Culham et al., 2018). For our studies on the osmostress protective properties of homoectoine (Nagata, 2001), we used the well-known *E. coli* K-12 laboratory strain MG1655 (Blattner et al., 1997) and an isogenic set of mutant strains derived from MG1655 with defects in either ProP or ProU, or in both transport systems (Supplementary Table S1). We grew these strains for osmostress protection assays in a chemically defined medium (MMA) with glucose as the carbon source and 0.8 M NaCl to increase the osmolarity. This degree of osmotic stress prevented the growth of the parent *E. coli* strain MG1655 while the presence of 1 mM ectoine, 5-hydroxyectoine, homoectoine, and, as a control glycine betaine, provided effective osmostress protection. Among the tested compatible solutes, homoectoine afforded the weakest degree of osmostress resistance, while ectoine, 5-hydroxyectoine, and glycine betaine rescued growth at high salinity to a similar extent (Figure 3A). Cells grown in the presence of homoectoine possessed a substantially longer lag-phase than those cultures that had received glycine betaine, ectoine, or 5-hydroxyectoine. During exponential growth, the cultures had a doubling time of approximately 2.5 h (for glycine betaine), 4 h (for ectoine and 5-hydroxyectoine), and 7.3 h (for homoectoine). The corresponding growth rates (μ) were as follows: μ = 0.27 h^–1^ for glycine betaine, μ = 0.17 h^–1^ for ectoine and 5-hydroxyectoine, and μ = 0.09 h^–1^ for homoectoine (Figure 3A).

In a next step of our analysis of the osmostress protective potential of homoectoine, we raised the salinity of the growth medium from 0.8 M NaCl to 1 M NaCl and found that homoectoine no longer conferred osmostress protection (Figure 3B). This elevated level of salinity in the growth medium also led to a differentiation of the osmostress protective attributes of ectoine, 5-hydroxyectoine, and glycine betaine that is reflected by the growth rates of these cultures: μ = 0.16 h^–1^ for glycine betaine, μ = 0.10 h^–1^ for 5-hydroxyectoine, and μ = 0.08 h^–1^ for ectoine. Hence, glycine betaine conferred the strongest level of osmostress protection in the *E. coli* strain MG1655, followed by 5-hydroxyectoine and ectoine (Figure 3B). Collectively, the data from these osmostress protection growth assays show that the synthetic ectoine derivative homoectoine is a moderately effective osmostress protectant, a conclusion that has previously also been reached by Nagata (2001). However, the *E. coli* W strain ATCC 9637 used by this author can withstand higher levels of salinity than the *E. coli* K-12 strain MG1655, since homoectoine was still osmostress protective in a minimal medium containing 1–1.2 M NaCl (Nagata, 2001).

The uptake route(s) for homoectoine in *E. coli* are unknown but since ectoine and 5-hydroxyectoine are imported *via* ProP and ProU (Jebbar et al., 1992), we suspected that these compatible solute importers with a broad substrate profile would also mediate the uptake of homoectoine. To test this, we constructed an isogenic set of *E. coli* MG1655 derivatives in which either the ProP or the ProU systems were operational, or in which both transport systems were simultaneously not functional. Osmostress protection growth assays confirmed that the ProP and ProU transporters were each proficient in homoectoine import, while their simultaneous genetic inactivation prevented its uptake. As expected from previous data, the same pattern was also observed for ectoine, 5-hydroxyectoine, and glycine betaine (Jebbar et al., 1992; Lucht and Bremer, 1994; Haardt et al., 1995) (Figure 3C).

### Docking of Homoectoine Into the Active Site of the Ectoine Hydroxylase EctD

The crystal structure of the ectoine hydroxylase from *S. alaskensis* in complex with the reaction product 5-hydroxyectoine, the catalytically important iron, and the co-substrate 2-oxoglutarate has recently been determined (PDB accession code: 4Q5O) (Höppner et al., 2014) (Figures 2A,B). This provided us with the opportunity to evaluate the suitability of the EctD active site to potentially accept the seven-membered homoectoine ring as a substrate for a hydroxylation reaction. The overall fold and dimeric assembly of the (*Sa*)EctD protein is shown in Figure 2A. The natural reaction product of the ectoine hydroxylase, (4*S*,5*S*)-5-hydroxyectoine, is bound within the active site through the coordination by the residues Gln-127, His-144, Thr-149, Trp-150, Arg-280, and Leu-284 (Höppner et al., 2014) (Figure 2B). The size of the (*Sa*)EctD enzyme reaction chamber was calculated with the CASTp program (Tian et al., 2018); it has a volume of approximately 77 Å^3^. Using *in silico* docking analysis with the programs Phenix, COOT, and AutoDock (Emsley and Cowtan, 2004; Trott and Olson, 2010; Afonine et al., 2012), we modeled the non-natural substrates for EctD, homoectoine, and its potentially hydroxylated derivative, hydroxyhomoectoine, into the active site of (*Sa*)EctD (Figures 2C,D). Both synthetic ectoine derivatives fit into the active site of the ectoine hydroxylase and might be coordinated by the same network of amino acid residues that also coordinate the native reaction product 5-hydroxyectoine (Höppner et al., 2014). As revealed by the docking approach, the spatial positioning of homoectoine and its hydroxylated derivative in the reaction chamber are super-imposable with that of the 5-hydroxyectoine molecule trapped in the (*Sa*)EctD crystal structure (PDB accession code: 4MHR) (Höppner et al., 2014) (Supplementary Figure S2A). Furthermore, our modeling study suggests that homoectoine could be positioned in the active site of the ectoine hydroxylase such that the position C5 of the expanded seven-member diazepine ring would potentially be modified with a hydroxyl group (Figures 2C,D).

### Enzyme Kinetics of Ectoine Hydroxylases From *P. stutzeri* A1501 and *S. alaskensis* Using Homoectoine as a Synthetic Substrate

**FIGURE 4:**
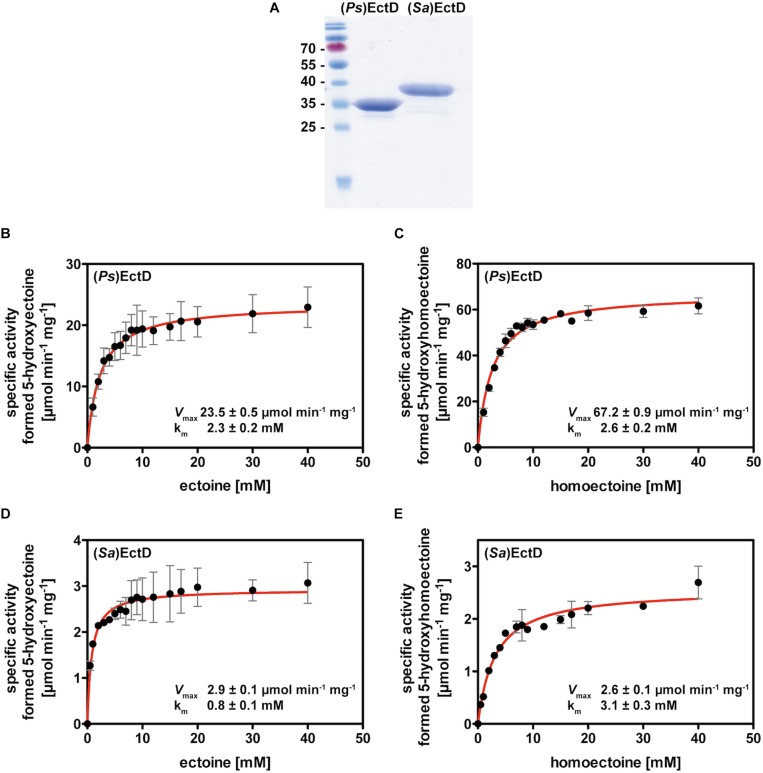
Enzyme kinetics using recombinantly produced and affinity purified EctD proteins from *P. stutzeri* A1501 and *S. alaskensis*. **(A)** SDS gel of purified ectoine hydroxylases. Shown is a 15% polyacrylamide SDS gel with the purified recombinant ectoine hydroxylases from *P. stutzeri* A1501 [(*Ps*)EctD] and *S. alaskensis* [(*Sa*)EctD]. The proteins were overexpressed in *E. coli* and purified *via Strep-Tag*-II affinity chromatography. 3 μg of both enzymes were applied to the gel and the pre-stained PageRuler Protein Ladder (Thermo Scientific) was used as a marker to size the migration of the two EctD proteins. **(B,C)** Kinetic parameters of the *P. stutzeri* A1501 (*Ps)*EctD and of the **(D,E)** *S. alaskensis*[(*Sa)*EctD] ectoine hydroxylases for the substrates **(B,D)** ectoine and **(C,E)** homoectoine.

To biochemically confirm the hypothesis that the EctD enzyme might promiscuously use the synthetic ectoine derivative homoectoine as a substrate, we conducted detailed kinetic analysis of the EctD proteins from *P. stutzeri* A1501 [(*Ps*)EctD] and *S. alaskensis* [(*Sa*)EctD]. These two proteins were chosen for further studies because a crystal structure of (*Sa*)EctD is available (Höppner et al., 2014) and the (*Ps*)EctD enzyme proved to be a highly efficient biocatalyst in an *E. coli* cell factory hydroxylating imported ectoine (Czech et al., 2016). Both recombinant proteins could be readily overproduced in *E. coli* and purified to apparent homogeneity as revealed by SDS-PAGE (Figure 4A). Despite a rather similar number of amino acids, calculated molecular weight [34.14 kDa for (*Sa*)EctD and 34.18 kDa for (*Ps*)EctD], and also pI [(*Sa*) EctD: 5.47, (*Ps*)EctD: 5.5], the electrophoretic mobility of the two proteins was notably different on a 15% SDS-PAGE (Figure 4A). This difference in electrophoretic mobility has previously been observed (Widderich et al., 2014a) but the underlying mechanism(s) remains unclear.

To conduct an analysis of the kinetic parameters of the (*Sa*)EctD and (*Ps*)EctD enzymes, we first optimized the reaction parameters from those of the previously used assay conditions (Höppner et al., 2014; Widderich et al., 2014a). In particular, we now used different temperatures for the two enzymes [15°C for (*Sa*)EctD and 35°C for (*Ps*)EctD], shortened the incubation time (5 min) of the assays to ensure linear turn-over during the enzyme reaction, reduced the protein concentration from 15 down to 1.47 μM, and substantially increased (fourfold) the concentration of the buffer [100 mM TES (pH 7.5)] but we kept the concentration of FeSO_4_ (1 mM), 2-oxoglutarte (10 mM), and KCl (100 mM) in the assay buffers identical to the previously used concentrations.

During the reaction of non-heme-containing iron(II) 2-oxoglutarate-dependent enzymes (Aik et al., 2012; Hangasky et al., 2013; Herr and Hausinger, 2018), reactive oxygen species can be produced and catalase is thus frequently used to mitigate their damaging effects. In test assays, the performance of the (*Ps*)EctD enzyme was not enhanced by the addition of catalase (up to 2500 U), while the performance of (*Sa*)EctD greatly benefitted from the addition of this H_2_O_2_ detoxifying enzyme. Hence, 1300 U of bovine liver catalase was added to the enzyme reactions when we assessed the kinetic parameters of the (*Sa*)EctD protein but did not add catalase to enzyme assays conducted with the (*Ps*)EctD protein. The expected reaction products of the ectoine hydroxylase, 5-hydroxyectoine as the authentic product and hydroxyhomoectoine as the predicted synthetic reaction product, were detected and quantified by HPLC analysis using a UV-detector set to 210 nm. Both ectoine and 5-hydroxyectoine (Bursy et al., 2007; Czech et al., 2016), and homoectoine and hydroxyhomoectoine (this study) could be cleanly separated on a GROM-SIL Amino-100 PR column (3 μm) (Supplementary Figure S3).

Under the optimized assay conditions and using ectoine as the substrate, the (*Sa*)EctD enzyme had a *k*_*m*_ of 0.8 ± 0.1 mM and a *V*_*max*_ of 2.9 ± 0.1 μmol formed 5-hydroxyectoine min^–1^ mg^–1^ protein, while the (*Ps*)EctD enzyme possessed a *k*_*m*_ of 2.3 ± 0.2 mM and a *V*_*max*_ of 23.5 ± 0.5 μmol formed 5-hydroxyectoine min^–1^ mg^–1^ protein (Figures 4B,D). The *V*_*max*_ of the (*Sa*)EctD enzyme for the synthetic substrate homoectoine matched that for its natural substrate, but this protein exhibited an about fourfold lower affinity for homoectoine (Figure 4E). While the *k*_*m*_ values of the (*Ps*)EctD enzyme for ectoine and homoectoine were very similar, the *V*_*max*_ for homoectoine substantially exceeded that for its natural substrate ectoine (by about threefold) (Figures 4B,C). Hence, both tested EctD enzymes are able to use the synthetic compound homoectoine as a substrate in a presumed hydroxylation reaction, in which the (*Ps*)EctD enzyme is apparently particularly effective.

### *In vivo* Hydroxylation of Homoectoine

**FIGURE 5:**
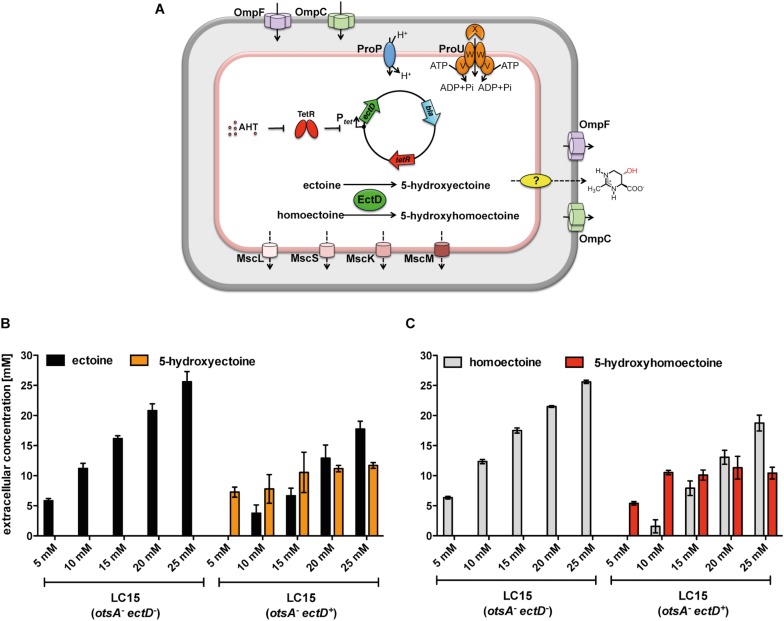
Production of 5-hydroxyectoine or 5-hydroxyhomoectoine from ectoine or homoectoine in an *E. coli* strain expressing the (*Ps*)EctD enzyme. **(A)** Schematic overview of the *E. coli* based EctD overproducing cell factory. The *E. coli* strain LC15 (*otsA*:Tn*10*) contained either the empty vector pASK-IBA3 (control) or the plasmid pMP41 (*ectD* gene from *P. stutzeri* A1501) and was incubated in the presence of various concentrations of **(B)** ectoine or **(C)** homoectoine (ranging from 5 to 25 mM). The cultures were incubated for 24 h after induction of *ectD* expression with AHT in baffled flasks containing 10 ml of MMA containing 0.4 M NaCl. The concentrations of **(B)** ectoine/5-hydroxyectoine and **(C)** homoectoine/5-hydroxyhomoectoine within the supernatant were determined *via* HPLC analysis. The data shown represent the means and standard deviations of at least three independently grown cultures (two independently grown cultures for controls).

*In vivo* biotransformation reactions to specifically hydroxylate chiral compounds using recombinant whole-cell biocatalysis are an environmentally friendly alternative to classical chemical synthetic procedures (Zhao et al., 2017). We have previously used such an approach to hydroxylate exogenously provided, and hence imported, ectoine in an *E. coli* cell factory expressing various plasmid-encoded *ectD* genes. In the recombinant *E. coli* strain, the newly formed 5-hydroxyectoine is continuously secreted/released into the growth medium (Czech et al., 2016). Building on these data, we fed various concentrations of homoectoine to a derivative of the *E. coli* K-12 strain MG1655 unable to synthesize its main osmostress protectant trehalose due to the presence of a *otsA:*Tn*10* insertion mutation (Hengge-Aronis et al., 1991; Strom and Kaasen, 1993), but expressing the *P. stutzeri ectD* gene under the control of the TetR-responsive *tet* promoter present on the plasmid pMP41 (Figure 5A). We grew these cells in MMA with a moderately increased salinity (0.4 M NaCl) to trigger enhanced expression of *proP* and *proU* in order to stimulate the uptake of ectoines *via* the ProP and ProU transport systems (Jebbar et al., 1992; Lucht and Bremer, 1994). In these experiments, we monitored the disappearance of homoectoine and the appearance of its presumed hydroxylated derivative in the growth medium by HPLC analysis. EctD-mediated biotransformation of ectoine into 5-hydroxyectoine was used as a control (Czech et al., 2016). When the empty expression vector (pASK-IBA3) was present in the *E. coli* MG1655 (*otsA*:Tn*10*) strain, as expected, no hydroxylated derivatives of either ectoine or homoectoine were detectable. The recovered amounts of ectoine and homoectoine closely matched those added to the growth medium at the beginning of experiments (Figures 5B,C). In contrast, when this experiment was carried out with the plasmid harboring the *P. stutzeri ectD* gene, hydroxylated derivatives of either ectoine or homoectoine appeared in the supernatant of the *E. coli* cultures (Figures 5B,C). Overall the *E. coli* K-12 strain MG1655 (*otsA*:Tn*10*) was only able to fully transform either 5 mM ectoine or 5 mM homoectoine into the corresponding hydroxylated derivatives (Figures 5B,C), while in a previous study, the *E. coli* K-12 strain MC4100 (*otsA*:Tn*10*) cell factory fully converted up to 15 mM ectoine into 5-hydroxyectoine (Czech et al., 2016). Thus, differences between even closely related *E. coli* K-12 laboratory strains seem to exist with respect to the efficiency in which they can import ectoines and convert them to the corresponding hydroxylated derivatives.

We observed that at substrate concentrations higher than 5 mM, a mixture of the originally added compound and its hydroxylated derivative was present in the growth medium. By adding up the sum of the substrate and the reaction product in the growth media, we found that the sum of ectoine and 5-hydroxyectoine after 24 h of incubation of the cells equaled the amount of initially added ectoine (Figure 5B). The calculated sum of homoectoine and hydroxyhomoectoine was always slightly higher than the initially added homoectoine concentration. We attribute these difficulties in quantification of hydroxyhomoectoine by HPLC analysis to its instability at neutral pH, complicating the exact calculation of the hydroxyhomoectoine content of samples from standard curves (Supplementary Figure S1).

### HPLC-MS and NMR Analysis Reveals Regio- and Stereoselective Hydroxylation of Homoectoine by the Ectoine Hydroxylase

So far, we have assumed that the product that we observe in the EctD-based *in vitro* and *in vivo* hydroxylation assays with homoectoine is actually hydroxyhomoectoine (Figures 4C,E, 5C and Supplementary Figure S3). To challenge this prediction, we used HPLC-MS (ESI +) to determine the molecular weight of the homoectoine-derived EctD reaction product. We used a supernatant of an *in vivo* biotransformation of an EctD cell factory that had received 5 mM homoectoine, so that the substrate had been completely imported, converted by the ectoine hydroxylase into the presumed hydroxyhomoectoine, which was then secreted (Figure 5C).

The supernatant of *E. coli* LC15 cells harboring the empty expression plasmid pASK-IBA3 (thereby lacking *ectD*) revealed a mass signal at 157.0970 *m/z* corresponding precisely to homoectoine with the chemical formula C_7_H_12_N_2_O_2_ and a calculated *m/z* of [M + H]^+^ = 157.0972 *m/z* (Supplementary Figure S4). The additionally observed signals at 179.0789 and 195.0529 *m/z* correspond to homoectoine molecules accompanied by either a Na^+^ or K^+^ ion instead of a proton. The supernatant of *E. coli* LC15 cells harboring the expression plasmid pMP41 (*ectD*^+^) revealed, however, a mass signal at 173.0917 *m/z* that corresponds precisely to hydroxyhomoectoine with the chemical formula C_7_H_12_N_2_O_3_ and a calculated mass of [M + H]^+^ = 173.0921 *m/z* (Supplementary Figure S4). Again, the additionally observed signals at 195.0738 and 211.0478 *m/z* correspond to hydroxyhomoectoine molecules in combination with a Na^+^ or K^+^ ion, respectively. HPLC-MS (ESI +) analysis of the MMA growth medium without cells and added homoectoine did not show any of the corresponding signals. Taken together, the HPLC-MS data thus unambiguously show that the (*Ps*)EctD enzyme can use the synthetic ectoine derivative homoectoine as a substrate and hydroxylate it.

**FIGURE 6:**
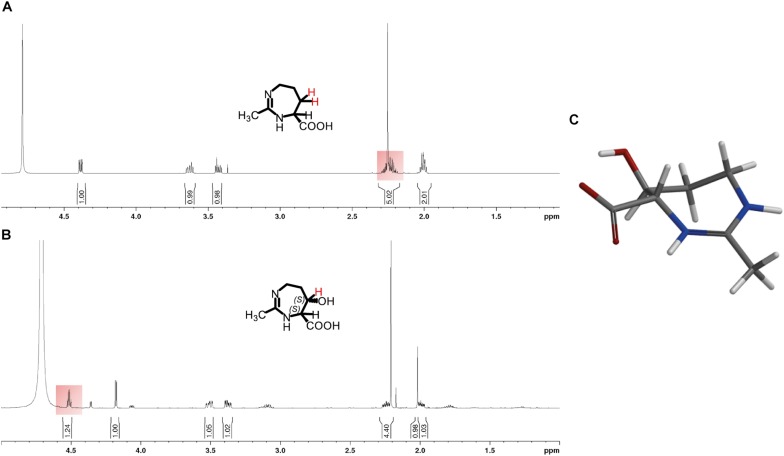
^1^H-NMR spectra of **(A)** homoectoine and **(B)** 5-hydroxyhomoectoine. The changing ^1^H-signal is highlighted in red. **(C)** DFT calculated structure of 5-hydroxyhomoectoine. Additional information and the recorded NMR spectra can be found in the Supplementary Material.

The ectoine hydroxylase is very precise in its natural enzymatic reaction, both with respect to its regio- and stereoselectivity. It is known to produce, both *in vivo* and *in vitro*, (4*S*,5*S*)-5-hydroxyectoine from its substrate ectoine (Inbar and Lapidot, 1988; Bursy et al., 2007). To assess if the EctD enzyme was also able to selectively hydroxylate the seven-membered diazepine ring of homoectoine, we used different types of NMR spectroscopy. For this purpose, we first purified the homoectoine derived hydroxylated compound *via* preparative HPLC from the supernatant of two 10-ml cultures of strain LC15 (pMP41-*ectD*^+^) that had received 5 mM homoectoine; this finally yielded 14 mg of the dried hydroxylated compound. After a quality check by HPLC-MS that ascertained that the isolated compound was actually hydroxyhomoectoine, the purified hydroxyhomoectoine was dissolved in water and its biosynthetic precursor homoectoine was dissolved in D_2_O (each in 700 μl) and were subsequently analyzed *via* 1d- and 2d-NMR. The comparison of ^1^H-, ^13^C, COSY-, HSQC-, and HMBC-spectra revealed exclusive hydroxylation at the C-5 position of the seven-membered homoectoine ring (Figure 6 and Supplementary Figures S5–S16). The signals of the NOE-spectrum were assigned to the DFT-calculated structure of (4*S*,5*S*)-hydroxyhomoectoine, which fits with a *trans*-configuration of the introduced hydroxyl group by the EctD enzyme (Figure 6C). The coupling constant between the α-H and the β-H of 5.46 Hz corresponds to a dihedral angle of approximately 38° by the Karplus equation (Karplus, 1959) which fits with a *trans*-configuration of the introduced hydroxyl group by the EctD enzyme (see Supplementary Material and Figure 6C). These data are fully consistent with the stereo-chemical configuration of the hydroxyl-group in 5-hydroxyectoine isolated from microorganisms and produced *in vitro* by the ectoine hydroxylase (Inbar and Lapidot, 1988; Bursy et al., 2007) (Figure 1). Furthermore, they nicely match the data from the modeling study predicting that EctD would hydroxylate the C-5 atom in the seven-membered diazepine ring of homoectoine (Figures 2C,D).

### 5-Hydroxyhomoectoine Is an Osmoprotectant

**FIGURE 7:**
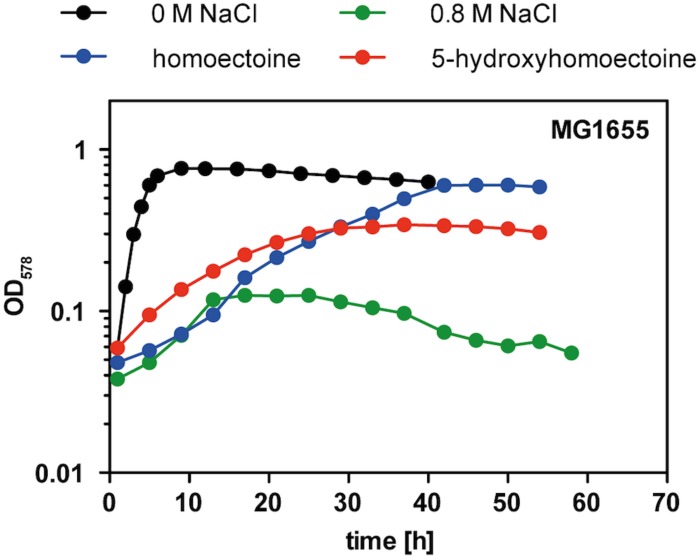
Osmostress protection assay with 5-hydroxyectoine and 5-hydroxyhomoectoine. Growth of *E. coli* MG1655 (*proU*^+^ *proP*^+^) in MMA without NaCl and in MMA containing 0.8 M NaCl without and with the addition of 1 mM of the indicated compatible solutes was monitored for 60 h. The OD_578_ of the cultures was measured hourly in a plate reader (Epoch2, BioTek). The osmostress protection growth assay was conducted with two independently grown cultures. A representative set of growth curves is shown.

Having the purified 5-hydroxyhomoectoine in hand, we tested its biological activity in an osmostress protection growth assay in a micro-titer well plate reader. Cells of *E. coli* MG1655 were grown under growth-restricting osmotic conditions (MMA with 0.8 M NaCl) in the absence or presence of various compatible solutes. As observed before (Figure 3A), homoectoine was a moderately effective osmostress protectant (Figure 7). 5-hydroxyhomoectoine was also an osmostress protectant but its effectiveness was even weaker than that of homoectoine (Figure 7). This could potentially be attributed to reduced import, the physico-chemical attributes of the compound itself, or reduced stability of 5-hydroxyhomoectoine in the pH-neutral MMA growth medium. We know that this latter effect plays a role because we observed a decrease in the slope of our standard curves over time when the samples prepared from the same stock solutions were re-measured (Supplementary Figure S1).

### 5-Hydroxyhomoectoine Is Released From *E. coli* Independent of Mechanosensitive Channels

The release of recombinantly produced ectoines from microorganisms that do not naturally synthesize these compounds has been repeatedly observed (Schubert et al., 2007; Zhang et al., 2009; Becker et al., 2013; Eilert et al., 2013; Ning et al., 2016). In previous reports, we have shown that newly synthesized ectoine and 5-hydroxyectoine are released from a recombinant *E. coli* strain independently of the ectoine importers ProP and ProU (Czech et al., 2018b). Furthermore, 5-hydroxyectoine was released from an EctD-producing *E. coli* strain in the absence of any currently known mechanosensitive channels (Edwards et al., 2012; Czech et al., 2016). These types of channels typically function as emergency release valves that allow the rapid and non-specific jettison of low-molecular-weight organic and inorganic solutes from suddenly osmotically down-shocked microbial cells (Booth, 2014; Cox et al., 2018). There is circumstantial evidence for the existence of a compatible solute efflux system in *E. coli* that potentially could also serve for the release of ectoine (Jebbar et al., 1992; Lamark et al., 1992) but its molecular identity and mode of action are unknown.

To assess if mechanosensitive channels were involved in the observed release/excretion of hydroxyhomoectoine (Figure 5C), we used an *E. coli* mutant that lacks all of the so far identified seven mechanosensitive channel genes (Edwards et al., 2012). The amount of hydroxyhomoectoine released from this mutant strain (MJF641) closely matched the amount released by its parental strain FRAG1 in which all mechanosensitive channels are intact (Supplementary Figure S17), as also observed for 5-hydroxyectoine produced from imported ectoine (Supplementary Figure S17). Consequently, these data exclude the involvement of any known mechanosensitive channel in the release of synthetically produced hydroxyhomoectoine by *E. coli*.

## Discussion

Microbial cells possess an “underground metabolism” originating from the promiscuous use of different substrates in side reactions of enzymes (D’Ari and Casadesus, 1998). This sloppiness of extant enzymes is an engine for the evolution of novel metabolic traits and can also be exploited for biotechnological purposes. The data that we report here focus on the substrate profile of the ectoine hydroxylase EctD (Bursy et al., 2007; Höppner et al., 2014), an enzyme that performs a precise regio- and stereoselective introduction of a hydroxyl group to an inactivated carbon within the chiral compound ectoine (Bursy et al., 2007; Widderich et al., 2014b). It endows the newly formed 5-hydroxyectoine (Inbar and Lapidot, 1988) with novel stress-protective and function preserving properties (Pastor et al., 2010; Czech et al., 2018a). Hence, the idea arose to exploit possible biosynthetic side activities of the ectoine hydroxylase as a catalyst in synthetic chemistry (Galinski et al., 2009; Hara et al., 2019). An attractive starting molecule for this approach is the synthetic ectoine derivative homoectoine (Schnoor et al., 2004) (Figure 1). It has already been shown to function as a superior PCR enhancer, and animal experiments suggest that it can potentially ameliorate the negative consequence of colitis better then ectoine by maintaining intestinal mucosal integrity (Schnoor et al., 2004; Shi and Jarvis, 2006; Castro-Ochoa et al., 2019; Farre and Vicario, 2019).

Molecular docking of homoectoine into the cavity of the cupin barrel of the *S. alaskensis* EctD protein, from which a high-resolution crystal structure is available (Höppner et al., 2014) (Figures 2A,B), suggested that the expanded seven-membered diazepine ring of homoectoine would not only nicely fit into the active site but that the ectoine hydroxylase would also introduce a hydroxyl group into the ring structure of this molecule at position C-5 (Figures 2C,D). HPCL-MS and various types of NMR analyses verified our *in silico* prediction when the *P. stutzeri* EctD enzyme was used in an *E. coli* cell factory to produce the hydroxylated derivate of homoectoine (Figures 5C, 6 and Supplementary Figures S4–S16). Collectively, our data show that the synthetic homoectoine molecule is modified in a very precise and regio- and stereoselective fashion to (4*S*,5*S*)-5-hydroxy-homoectoine in which the hydroxyl group is introduced in a *trans* configuration (Figure 1).

Some of the kinetic parameters that we measured for the EctD-mediated conversion of homoectoine into 5-hydroxyhomoectoine are rather surprising because the *V*_*max*_ of the (*Ps*)EctD enzyme for its synthetic substrate homoectoine exceeds that for its natural substrate ectoine by about threefold (Figures 4B,C). The (*Ps*)EctD enzyme is also considerably more efficient in converting both ectoine and homoectoine into the corresponding hydroxylated species than the (*Sa*)EctD ortholog (Figure 4). The superior performance of the (*Ps*)EctD enzyme was already noted when several EctD enzymes were benchmarked against each other in a recombinant *E. coli* strain producing 5-hydroxyectoine from imported ectoine (Czech et al., 2016). However, this observation could not be properly explained from the previously determined kinetic parameters obtained under assay conditions somewhat different from those used here (Widderich et al., 2014a).

The ectoine hydroxylase was originally regarded as a highly specific enzyme because the EctD proteins from *S. coelicolor* and *Salibacillus salexigens* apparently did not hydroxylate L-proline, and the *S. salexigens* enzymes were not active toward the synthetic ectoine derivatives DHMICA, a five-membered ring molecule, and homoectoine, a seven-membered ring molecule (Bursy et al., 2007, 2008). However, in hindsight, and taking the data from this study into account, these data now need to be viewed with some caution. It is not necessarily clear that these two EctD enzymes cannot perform the hydroxylation reaction using unusual substrates in general but rather additional assay optimization might be required to reveal the complete substrate profiles of the *S. coelicolor* and *S. salexigens* ectoine hydroxylases.

Hydroxylated prolines are interesting building blocks for medical and biotechnological applications as these can be incorporated in cyclic non-ribosomal peptide compounds, such as the antifungal agent echinocandin or the anti-tuberculosis drugs griselimycins (Houwaart et al., 2014; Lukat et al., 2017; Zhang et al., 2018). In a recent study, the EctD enzymes from *H. elongata* and *Streptomyces cattleya* were utilized to produce hydroxyprolines (Hara et al., 2019). While EctD from *H. elongata* only catalyzed the formation of *trans*-3-hydroxyproline from L-proline, the (*Sc*)EctD enzyme also accepted 3,4-dehydro-L-proline, 2-methyl-L-proline, and L-pipecolic acid as substrates, highlighting that notable differences in the substrate profiles and kinetic parameters (Figures 4B–D) of *bona fide* ectoine hydroxylases exist (Höppner et al., 2014; Widderich et al., 2014a; Czech et al., 2018a).

**FIGURE 8:**
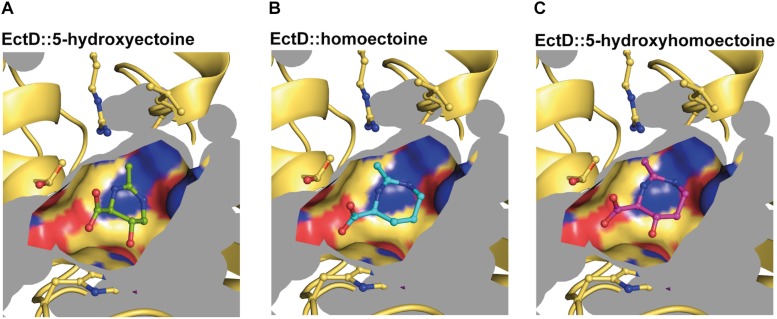
Modeling and docking of different substrates into the crystal structure of the ectoine hydroxylase EctD from *S. alaskensis.* The cavity of the active site of the *Sa*EctD enzyme (PDB accession code: 4Q5O) is shown with **(A)** the natural reaction product 5-hydroxyectoine, **(B)** the synthetic substrate homoectoine, and **(C)** the synthetic reaction product 5-hydroxyhomoectoine.

The (*Sa*)EctD enzyme reaction chamber has a calculated volume of approximately 77 Å^3^ (Figure 8). This is an important number that should be taken into account when larger non-natural substrates are considered for *in vivo* or *in vitro* EctD-mediated hydroxylation reactions. Models of the (*Sa*)EctD reaction chamber containing either the synthetic substrate homoectoine or the reaction products 5-hydroxyectoine and 5-hydroxyhomoectoine are shown in Figure 8. Our data on the docking analysis of homoectoine into the crystal structure of the (*Sa*)EctD enzyme (Figure 2C) suggest that such an *in silico* approach can probably generally serve to predict the hydroxylation site for non-natural substrates of the ectoine hydroxylase. As a proof of principle, the report by Hara et al. (2019) that the EctD enzyme from *H. elongata* displays selective *trans*-3-hydroxylation activity toward L-proline (Hara et al., 2019) motivated us to dock L-proline acid into the active site of the (*Sa*)EctD protein. Notably, this model predicted correctly the position at which the L-proline molecule is actually hydroxylated by the (*He*)EctD enzyme to form *trans*-3-hydroxyproline (Supplementary Figure S2B).

There are three important lessons that can be learned from our studies and the report of Hara et al. (2019). First, enzymatic assays with various ectoine hydroxylases might require specific optimization to perform optimally with the natural substrate ectoine and various non-natural substrates. Second, the use of EctD enzymes from different microorganisms can be helpful to benchmark their activities *in vitro* and *in vivo* against each other in order to identify the best suitable candidate for biotransformation reactions. Third, it is clear now that the enzymatic performance of EctD-type enzymes and their promiscuous use of secondary substrates (Galinski et al., 2009, 2015; Hara et al., 2019) (this study) are not *per se* deducible form their amino acid sequences, as these proteins are evolutionarily closely related (Supplementary Figure S18) and similar in their structural fold (Reuter et al., 2010; Höppner et al., 2014; Widderich et al., 2014a; Czech et al., 2018a).

Homoectoine and 5-hydroxyhomoectoine are moderately effective osmostress protectants (Nagata, 2001) (Figures 3A,B, 7). This could potentially be rooted in their physico-chemical attributes, or more likely, it may be that the *E. coli* ProU and ProP transport systems for ectoines (Jebbar et al., 1992; MacMillan et al., 1999; Czech et al., 2016) are not optimally configured to accommodate the seven-membered rings of these compounds. This points to a potentially serious limitation when EctD-based microbial cell factories will be used to hydroxylate non-natural substrates of the ectoine hydroxylase (Galinski et al., 2009, 2015). This might be overcome by the use of permeabilized cells (Galinski et al., 2009), as has been demonstrated for the recombinant synthesis of ectoine using the *H. elongata ectABC* genes in *E. coli* (He et al., 2015). The ectoine hydroxylase is a strictly oxygen- and 2-oxoglutarate-dependent enzyme (Höppner et al., 2014; Widderich et al., 2014a, b, 2016). Hence, insufficient availability of the co-factor 2-oxoglutarate and a limited oxygen supply could severely impede the maximal performance of EctD-based microbial cell factories. In case of the recombinant *E. coli ectD*^+^ strain producing *trans*-3-hydroxyproline, constraints on the supply of 2-oxoglutarate have been averted by deleting the gene for the 2-oxoglutarate-consuming 2-oxoglutarate dehydrogenase, thereby increasing the yield of *trans*-3-hydroxyproline by twofold (Hara et al., 2019). Limitations in the supply of oxygen were avoided in a synthetic cell factory expressing a proline-4-hydroxylase from *Dactylosporangium* sp. strain RH1 by implanting a hemoglobin gene from *Vitreoscilla* into the *E. coli* chromosome, thereby increasing production of the hydroxylated proline derivative by twofold (Zhao et al., 2017).

The ability to synthesize ectoine and hydroxylate it *via* EctD to 5-hydroxyectoine is widely found in members of ten major phyla of the *Bacteria*. 5-Hydroxyectoine-producing bacteria live in ecophysiologically rather varied habitats with respect to salinity, temperature, and pH, and they can also be found both in marine and terrestrial environments (Czech et al., 2018a). The ectoine hydroxylases from these microorganisms should therefore be a rich source to search for EctD proteins with biotechnologically interesting substrate profiles. Furthermore, the available EctD crystal structures (Reuter et al., 2010; Höppner et al., 2014) can be used as starting templates for targeted or high-throughput mutagenesis approaches to potentially improve the catalytic efficiency of the ectoine hydroxylase for its natural or synthetic substrates, or to broaden its substrate profile (Koketsu et al., 2015; Hara et al., 2019). If appropriate selection conditions can be designed, laboratory evolution experiments (Guzman et al., 2019) could also come into play to shape the catalytic performance and substrate profile of ectoine hydroxylases for uses in commercially interesting biotransformation reactions.

## Data Availability Statement

All datasets generated for this study are included in the article/Supplementary Material.

## Author Contributions

LC and EB conceived and directed this study. LC, SW, OC, UL, and JB conducted the experiments and evaluated the data. SS carried out modeling studies. LC, SS, EG, and EB wrote the manuscript. All authors commented on the manuscript.

## Conflict of Interest

The authors declare that the research was conducted in the absence of any commercial or financial relationships that could be construed as a potential conflict of interest.

## Footnotes

1 graphpad.com

2 https://gaussian.com/citation/

3 www.pymol.org/2/

4 snapgene.com

5 www.adobe.com/products/illustrator.html
