# Supplementary material for: Exploiting Substrate Promiscuity of Ectoine Hydroxylase for Regio- and Stereoselective Modification of Homoectoine
Source: Front Microbiol. 2019 Nov 27;10:2745. doi: 10.3389/fmicb.2019.02745 (PMC6890836; doi:10.3389/fmicb.2019.02745)
Supplement: Supplementary file 1 [file Data_Sheet_1.PDF]

Supplementary material for:

Exploiting substrate promiscuity of ectoine hydroxylase for regio- and stereoselective modification of homoectoine

Laura Czech[1], Sarah Wilcken[1], Oliver Czech[2], Uwe Linne[2], Jarryd Brauner[3], Sander H.J. Smits[4,5], Erwin A. Galinski[3], and Erhard Bremer[1,6]*

[1]Laboratory for Microbiology, Department of Biology, Philipps-University Marburg, Marburg, Germany
[2]Department of Chemistry, Philipps-University Marburg, Marburg, Germany
[3]Institute of Microbiology and Biotechnology, Rheinische Friedrich-Wilhelms-University, Bonn, Germany
[4]Institute of Biochemistry, Heinrich-Heine University Düsseldorf, Düsseldorf, Germany
[5]Center for Structural Studies, Heinrich-Heine University Düsseldorf, Düsseldorf, Germany
[6] SYNMIKRO Research Center, Philipps-University Marburg, Marburg, Germany

Running title: Chemical biology of ectoines

Number of figures: 18

Number of tables: 2

For correspondence during the reviewing and editorial process please contact:

Dr. Erhard Bremer, Philipps-University Marburg, Dept. of Biology, Laboratory for Microbiology, Karl-von-Frisch-Str. 8, D-35032 Marburg, Germany. Phone: (+49)-6421-2821529. Fax: (+49)-6421-2828979. E-Mail: bremer@staff.uni-marburg.de

____

*Correspondence:

Erhard Bremer: bremer@staff.uni-marburg.de

Table S1 Strains used and constructed in this study.

Strain*	Genotype	Reference or source
BL21	*E. coli* B, F⁻ *ompT gal dcm lon hsdS*$_B$(*r*$_B$⁻*m*$_B$⁻) λ(DE3 [*lacI lacUV5-T7p07 ind1 sam7 nin5*]) [*malB*$^+$]$_{K\text{-}12}$(λ^S)	(Studier et al., 1990)
FRAG1	*E. coli* K-12, F⁻ *rha thi gal lacZ*	(Epstein and Kim, 1971)
MJF641	FRAG1 *mscS kefA*::*kan ybdG*::*apr ybiO yjeP ynaI ycjM*::Tn*10 mscL::cml*	(Edwards et al., 2012)
MG1655	*E. coli* K-12, F⁻ λ⁻ *ilvG rfb-50 rph-1*	(Blattner et al., 1997)
LC11	MG1655 (Δ*proU*::*spc*)*608* [*proP*$^+$]	This study
LC12	MG1655 (Δ*proP*::*kan*)*737* [*proU*$^+$]	This study
LC14	MG1655 (Δ*proU*::*spc*)*608* (Δ*proP*::*kan*)*737*	This study
LC15	MG1655 *otsA1::*Tn*10*	This study

*Strains LC11, LC12, and LC15 were constructed by transducing the *E. coli* strain MG1655 with a P1*vir* lysates prepared either on strain MKH17 (Δ*proU*::*spc*)*608* (Haardt et al., 1995), on strain JW4072-1 (*ΔproP737*::*kan*) (Baba et al., 2006), or on strain FF4169 (*otsA1*::Tn*10*) (Strom and Kaasen, 1993), respectively. Strain LC14 was constructed by transducing strain LC11 with the P1 lysate from strain JW4072-1 (*ΔproP737*::*kan*). Transductants were selected on LB agar plates containing the appropriate antibiotic.

Table S2 Plasmids used in this study.

Plasmid	Description	Reference
pASK-IBA3	Expression plasmid, C-terminal Strep-tag, tet_P, Amp^R	IBA GmbH, Göttingen
pMP40	pASK-IBA3 derivative with *ectD* from *S. alaskensis*, Amp^R	(Widderich et al., 2014)
pMP41	pASG-IBA3 derivative with *ectD* from *P. stutzeri*, Amp^R	(Widderich et al., 2014)

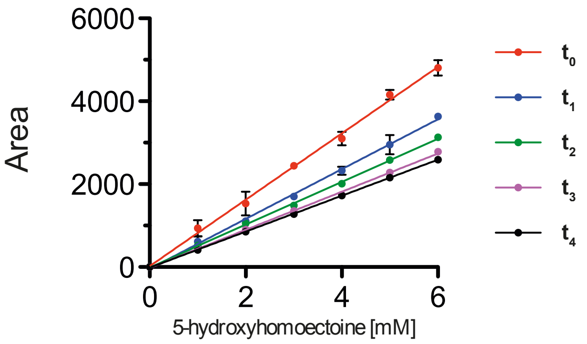

Supplementary Figure 1. Instability of 5-hydroxyhomoectoine standard solutions. Dilutions of purified 5-hydroxyhomoectoine were measured via HPLC to allow the determination of 5-hydroxyhomoectoine concentrations in the supernatants of *E. coli* cell factory expressing the *Pseudomonas stutzeri ectD* gene. The freshly prepared standard dilutions of 5-hydroxyhomoectoine were immediately stored at -20°C. t_0 indicated the fresh standard solution, while t_1, t_2, t_3, t_4 show the standard curves of 5-hydroxyhomoectoine after 2, 12, 13 and 15, weeks of storage at this temperature, respectively.

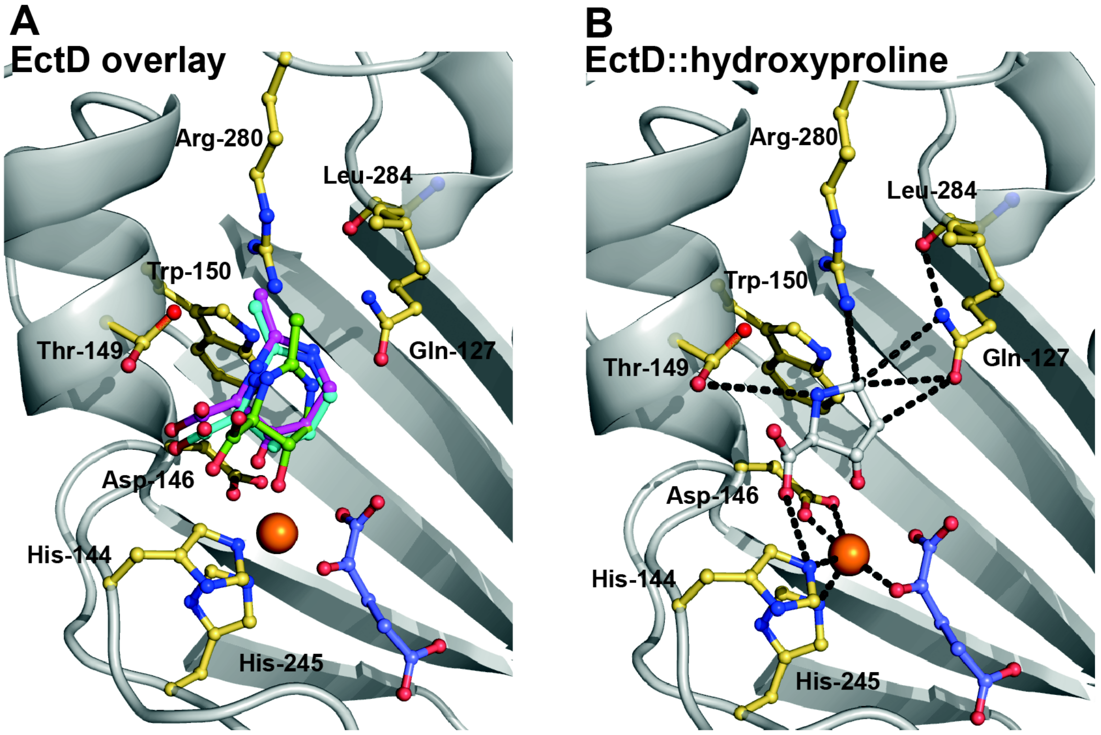

Supplementary Figure 2. Modeling and docking of different substrates into the crystal structure of the ectoine hydroxylase EctD from *S. alaskensis* (PDB: 4Q5O). **(A)** Zoom into the active site of one (*Sa*)EctD monomer with an overlay of different substrates. The natural reaction product of the ectoine hydroxylase, 5-hydroxyectoine, is shown in green, the modeled synthetic substrate homoectoine in blue, and the modeled reaction product of the EctD-catalyzed hydroxylation of homoectoine, 5-hydroxyhomoectoine, in pink. The co-substrate of the EctD enzyme, 2-oxoglutarate, is shown in light blue, and the catalytically important iron atom is depicted as an orange ball. **(B)** Zoom into the active site of one (*Sa*)EctD monomer bound to 3-hydroxyproline (grey). This non-natural substrate of the ectoine hydroxylase was modeled into the active side of the EctD enzyme. Amino acids involved in substrate binding are shown as yellow sticks and possible interactions are indicated by black dotted lines.

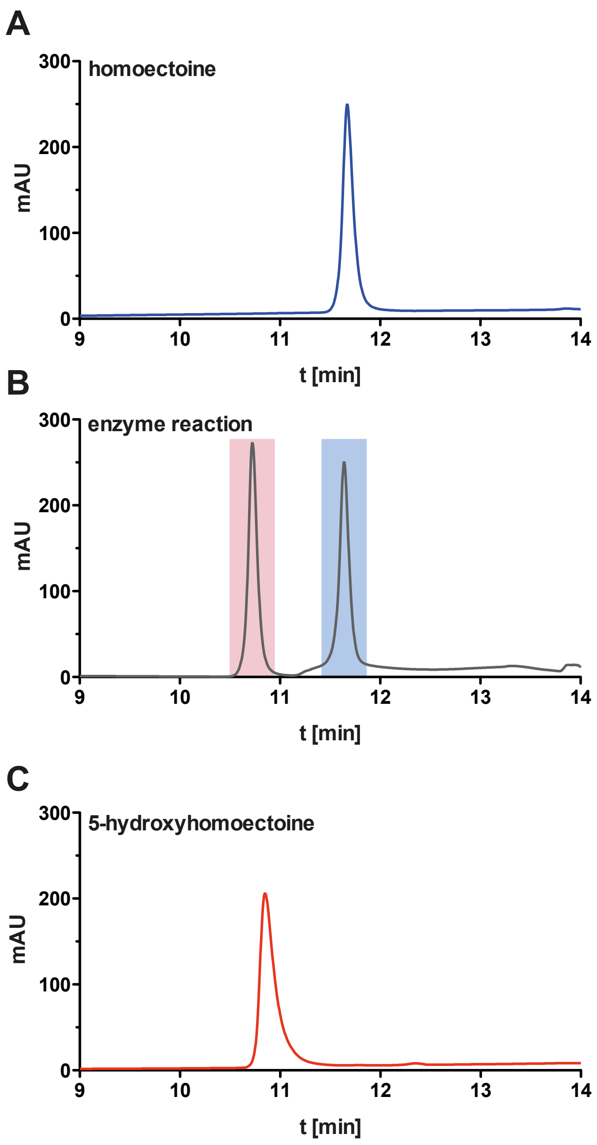

Supplementary Figure 3. HPLC chromatograms of **(A)** the homoectoine standard (blue), **(B)** a (*Ps*)EctD-catalyzed enzyme reaction with a mixture of the substrate homoectoine (blue) and the reaction product 5-hydroxyhomoectoine (red), and **(C)** the 5-hydroxyhomoectoine standard. Ectoines were detected at a wavelength of 210 nm (Kuhlmann and Bremer, 2002).

A

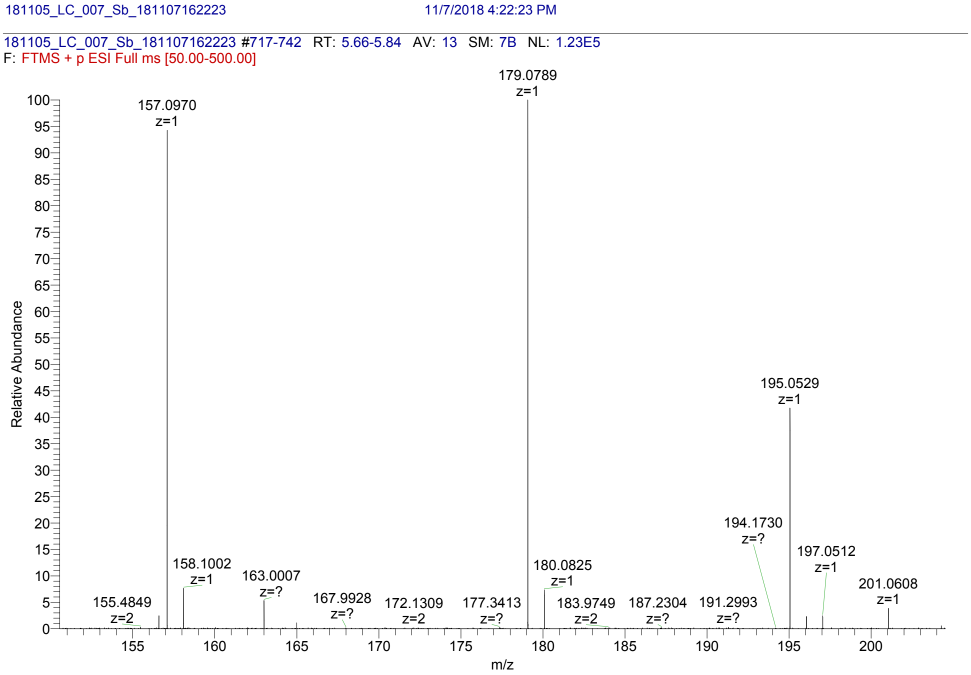

B

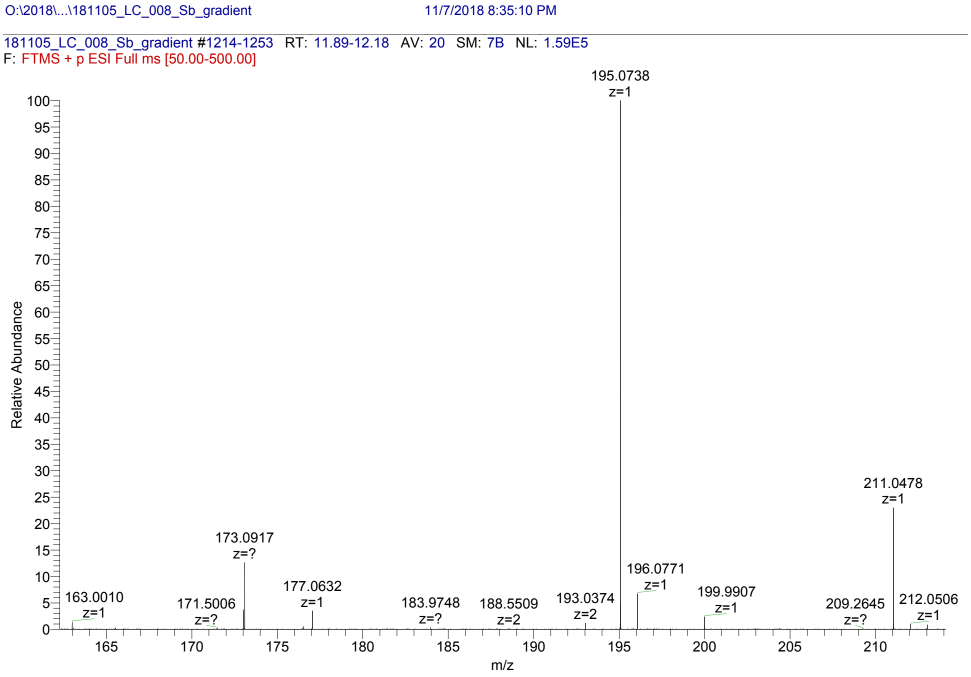

Supplementary Figure 4. Mass spectra of **(A)** homoectoine and **(B)** 5-hydroxyhomoectoine detected in the supernatant of an *E. coli* LC15 (*otsA*::Tn*10*) cell factory harboring either the empty vector **(A)** pASK-IBA3 or **(B)** expressing the (*Ps)*EctD enzyme from the plasmid pMP41 (*ectD* gene from *P. stutzeri* A1501). The calculated theoretical molecular mass of homoectoine is 157.0972 g/mol and 173.0921 g/mol for 5-hydroxyhomoectoine.

Supplemental information – NMR data

(S)-2-methyl-4,5,6,7-tetrahydro-1H-1,3-diazepine-4-carboxylic acid (homoectoine)

^{1}H-NMR (D_2O, 500.13 MHz) d = 1.98-2.03 (2H, m, 6-H), 2.21 (1H, ddd, J=6.02, 6.21, 8.85 Hz, 5-H_a), 2.25 (3H, s, 8-H), 2.27 (1H, ddd, J=3.39, 7.53, 14.68 Hz, 5-H_b), 3.43 (1H, ddd, J=5.37, 5.18, 14.81 Hz, 7-H_a), 3.63 (1H, ddd, J=6.37, 7.56, 14.09 Hz, 7-H_b), 4.39 (1H, dd, J=3.90, 8.76 Hz, 4-H).

^{13}C (75.49 MHz) d = 20.6 (C8), 24.5 (C6), 29.7 (C5), 43.4 (C7), 58.8 (C4), 164.9 (C2), 176.8 (COO^-).

(4*S*,5*S*)-5-hydroxy-2-methyl-4,5,6,7-tetrahydro-1*H*-1,3-diazepine-4-carboxylic acid (5-hydroxy-homoectoine)

^{1}H-NMR (H_2O, 600.23 MHz) d = 2.08 (1H, dddd, J=2.85, 4.61, 8.24, 15.80 Hz, 6-H_a), 2.29 (3H, s, 8-H), 2.32 (1H, dddd, J=2.78, 5.83, 9.75, 15.73 Hz, 6-H_b), 3.46 (1H, ddd, J=2.70, 8.46, 15.19 Hz, 7-H_a), 3.59 (1H, ddd, J=2.60, 9.77, 14.99 Hz, 7-H_b), 4.26 (1H, d, J=5.46 Hz, 4-H), 4.51 (1H, dt, J=5.46, 8.24 Hz, 5-H).

^{13}C (75.49 MHz) d =174.4 (COO^-), 166.0 (C2), 68.7 (C5), 64.1 (C4), 39.0 (C7), 31.9 (C6), 20.3 (C8).

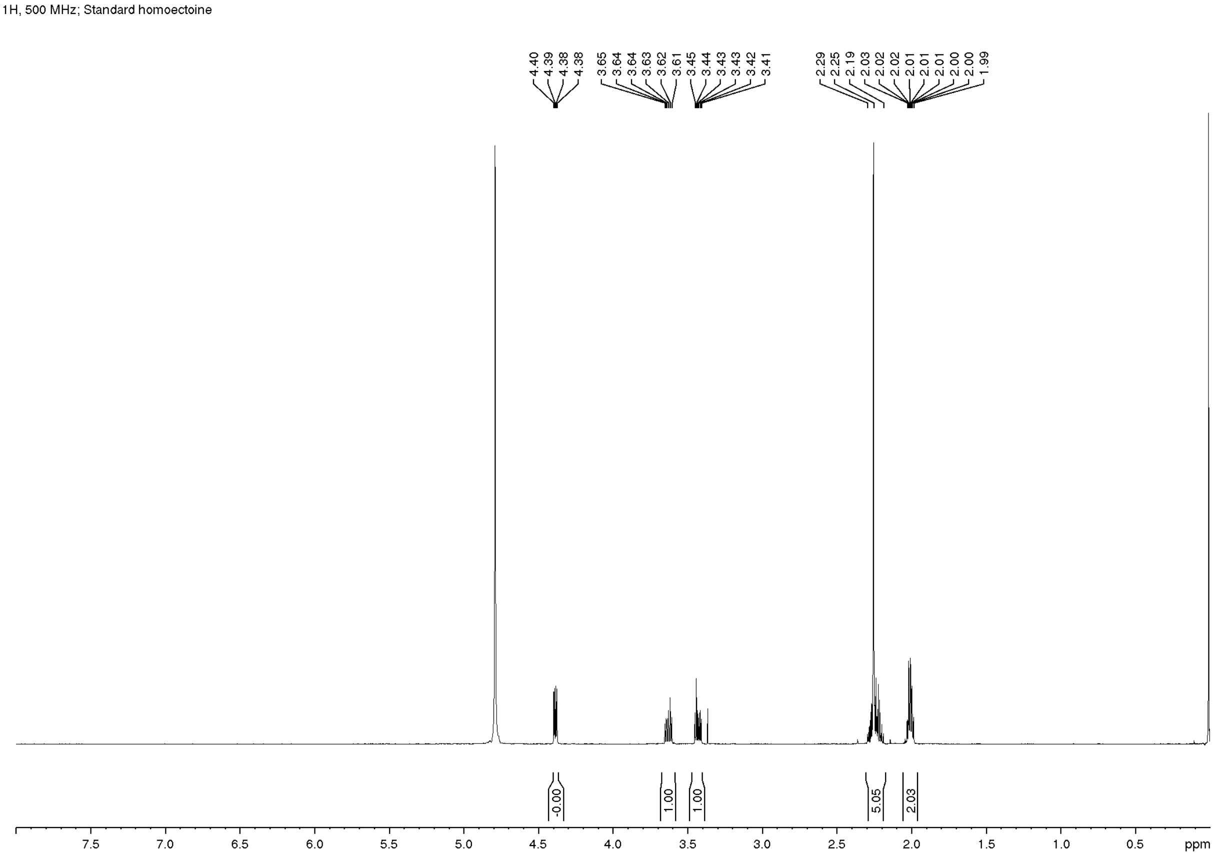

Supplementary Figure 5. ^{1}H NMR spectrum of homoectoine.

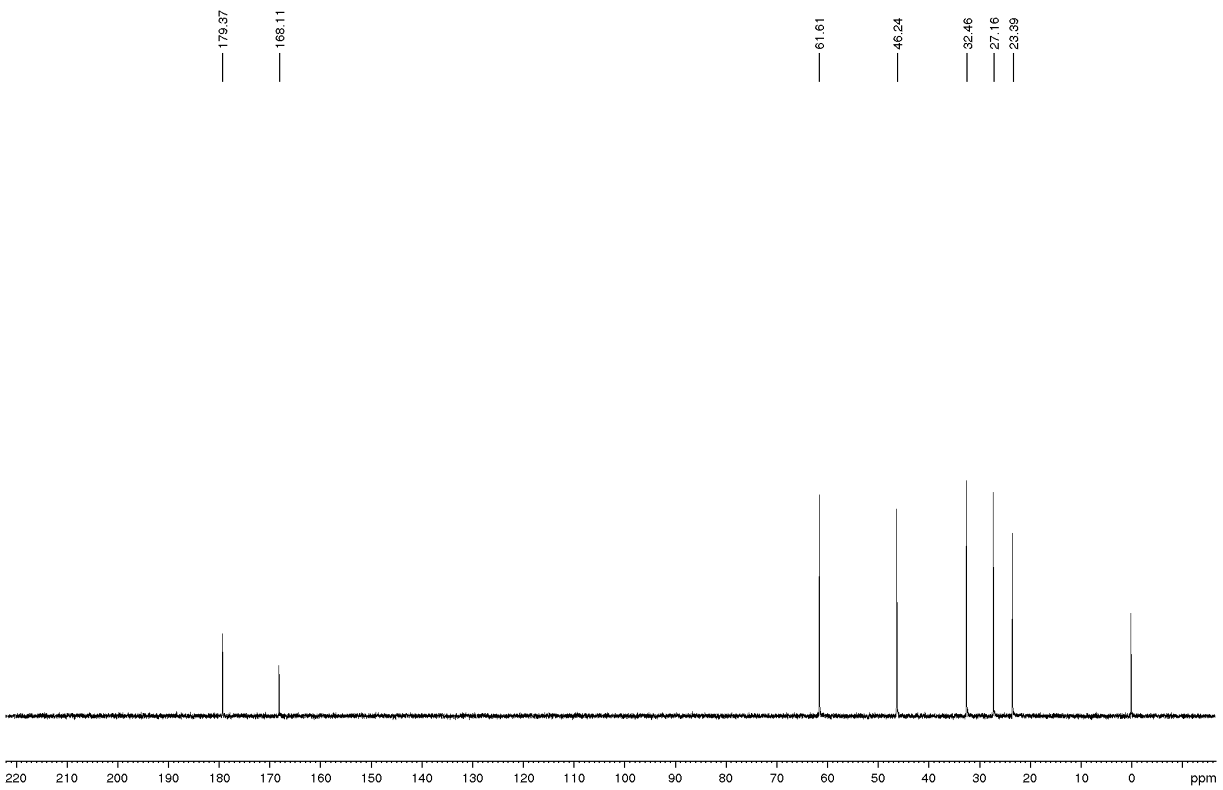

Supplementary Figure 6. ^{13}C NMR spectrum of homoectoine.

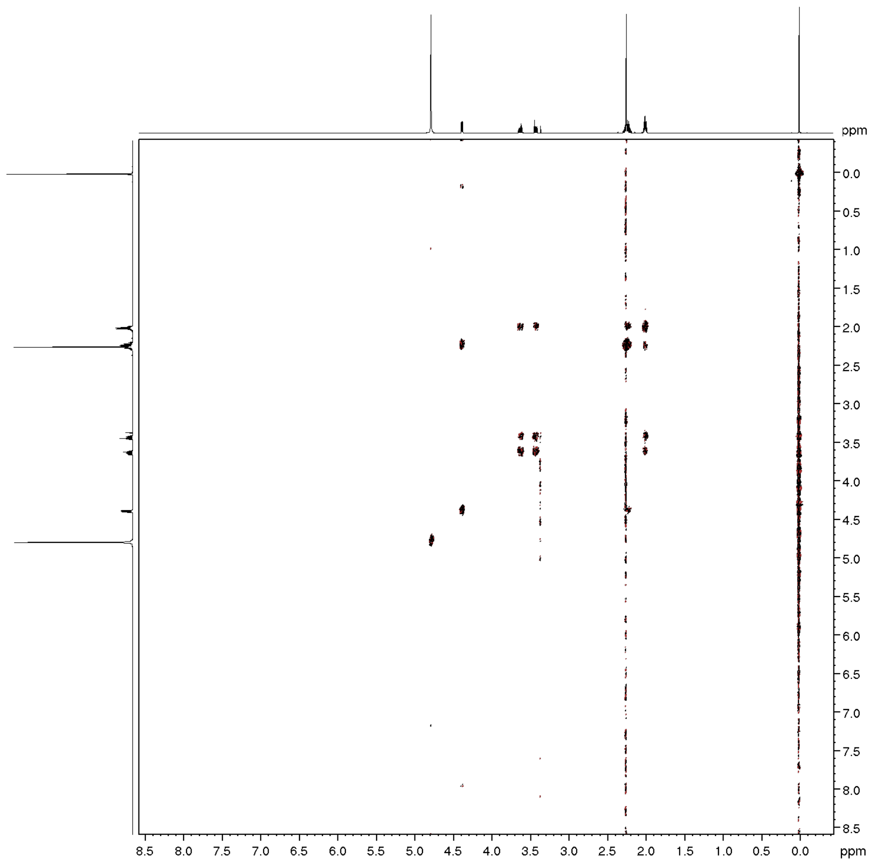

Supplementary Figure 7. COSY spectrum of homoectoine.

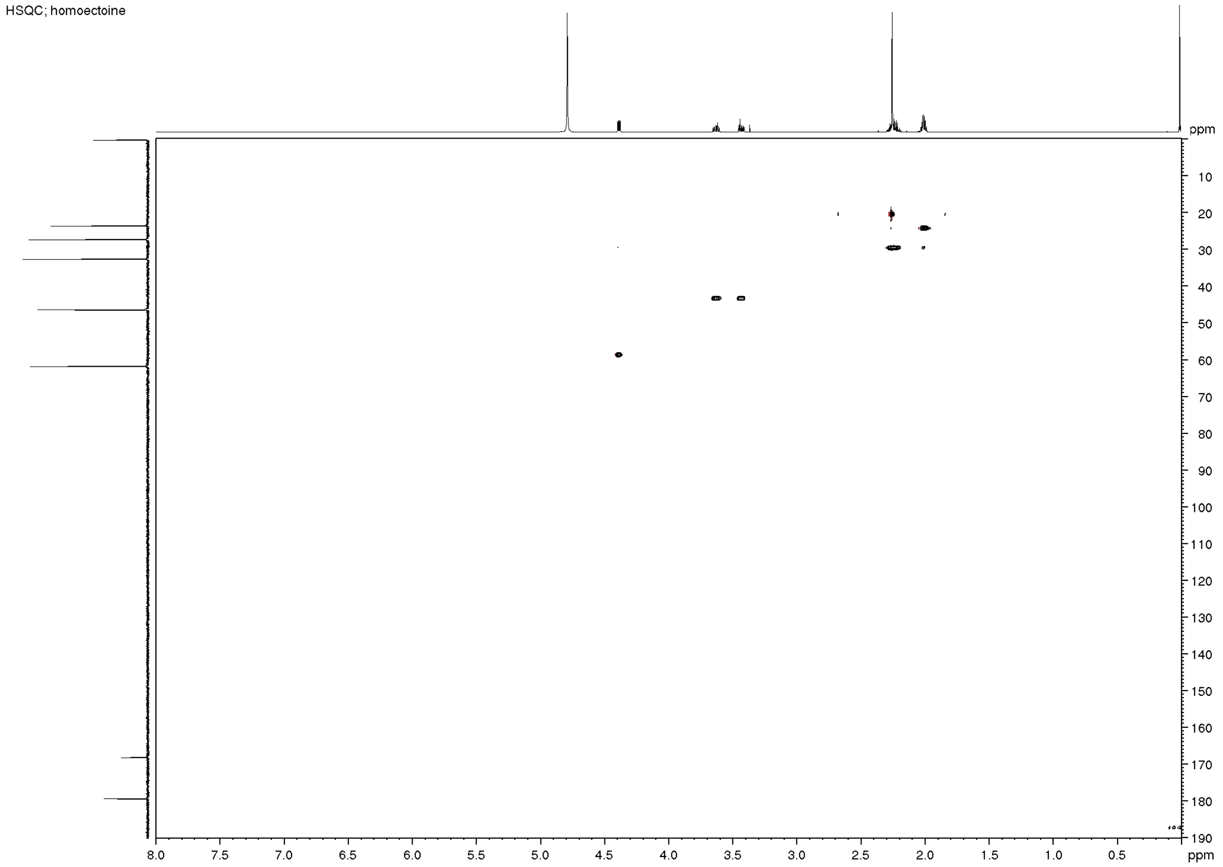

Supplementary Figure 8. HSQC spectrum of homoectoine.

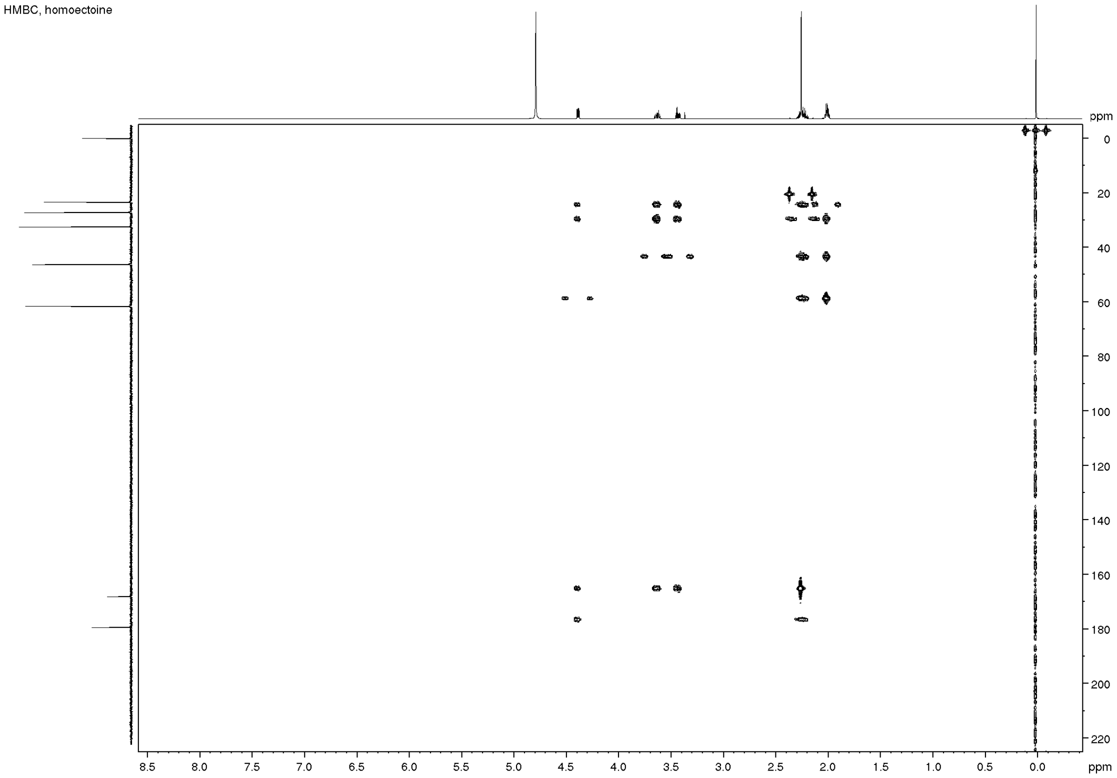

Supplementary Figure 9. HMBC spectrum of homoectoine.

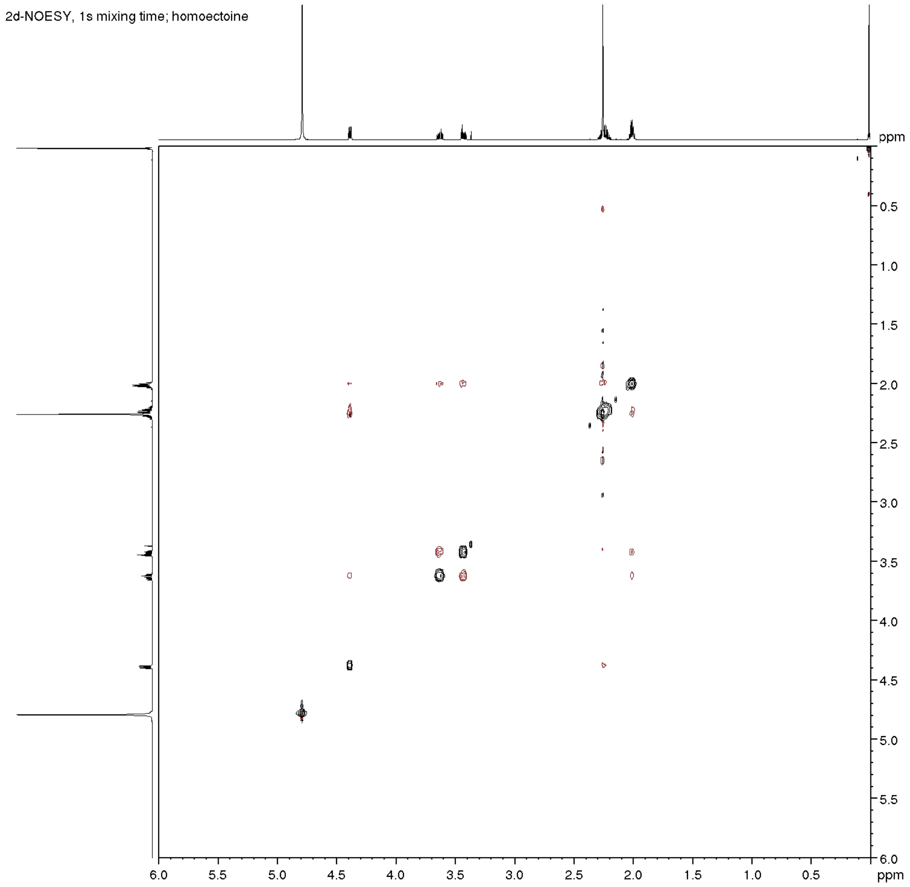

Supplementary Figure 10. NOESY spectrum of homoectoine.

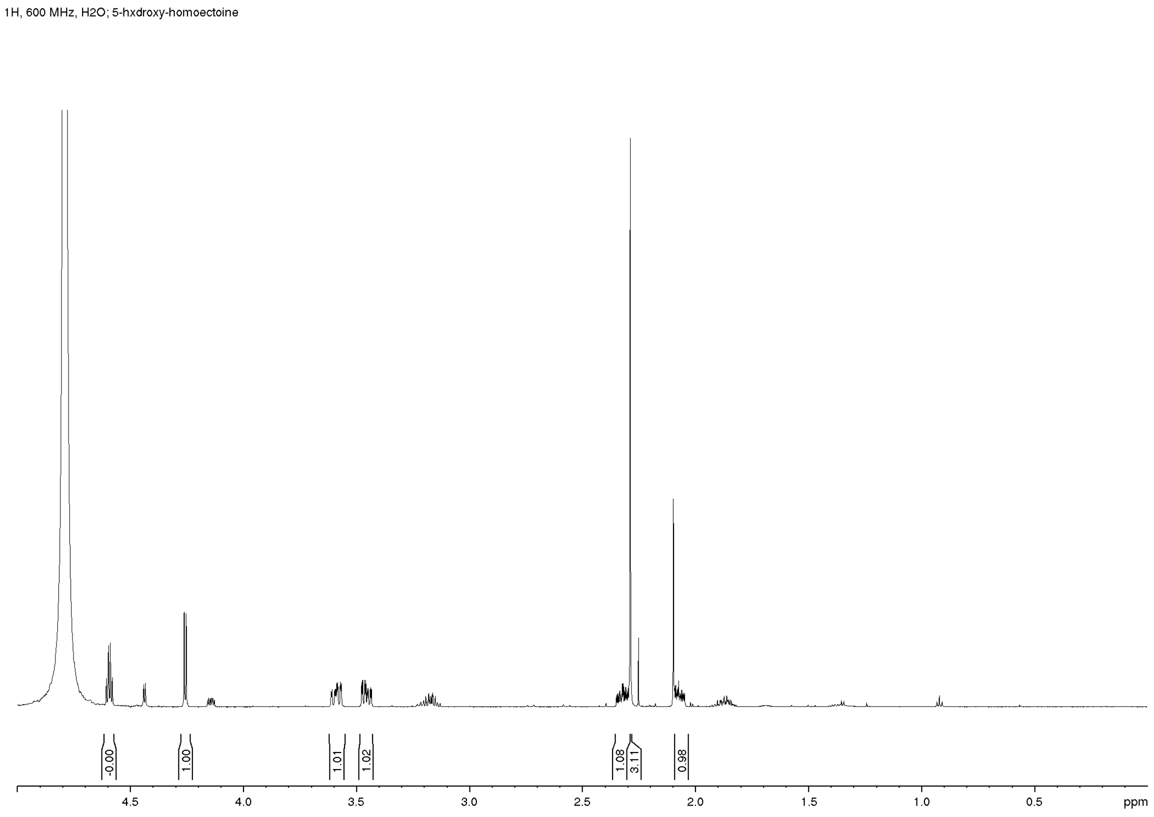

Supplementary Figure 11. ^{1}H NMR spectrum of 5-hydroxyhomoectoine.

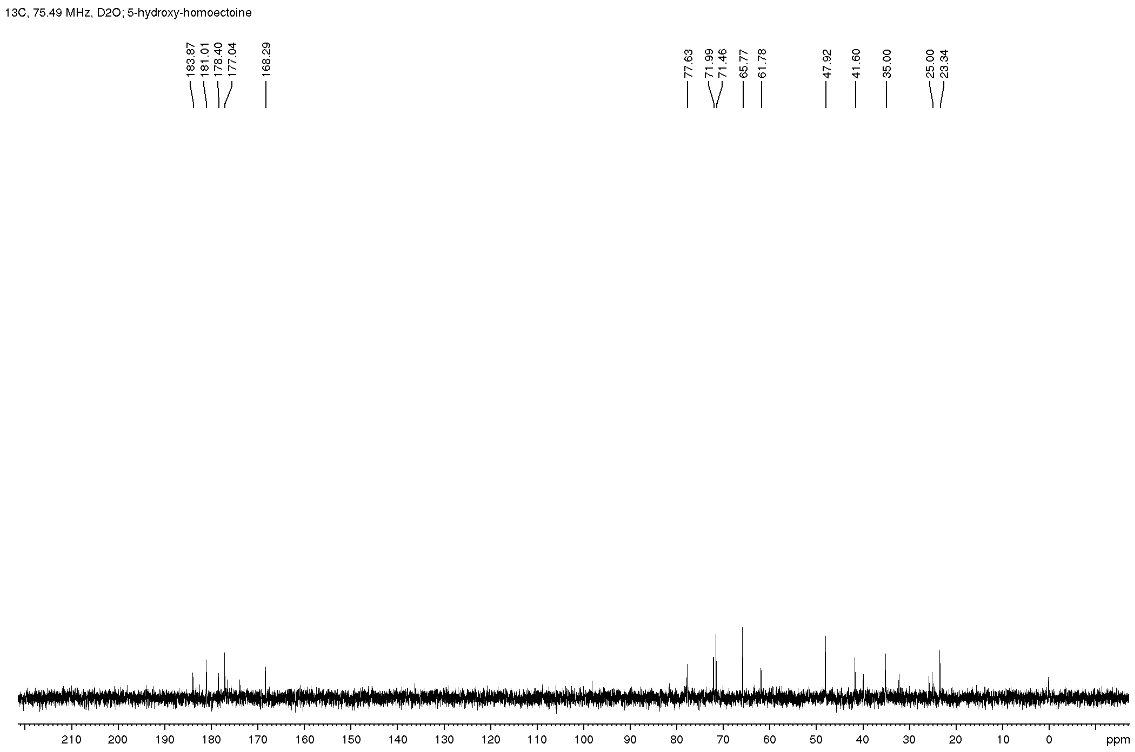

Supplementary Figure 12. ^{13}C NMR spectrum of 5-hydroxyhomoectoine.

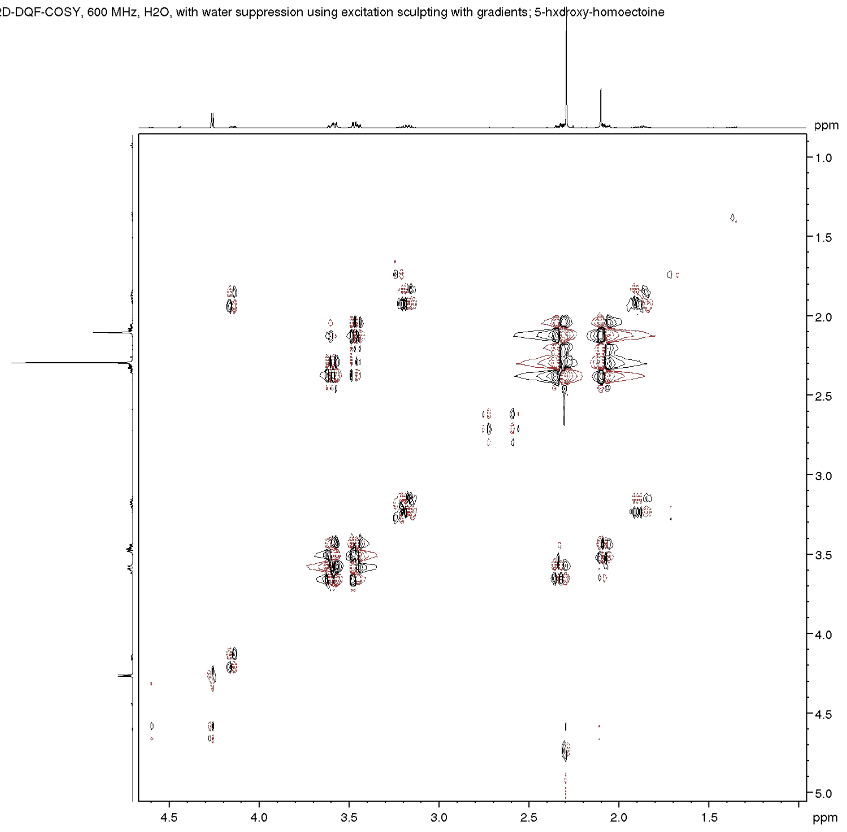

Supplementary Figure 13. COSY spectrum of 5-hydroxyhomoectoine.

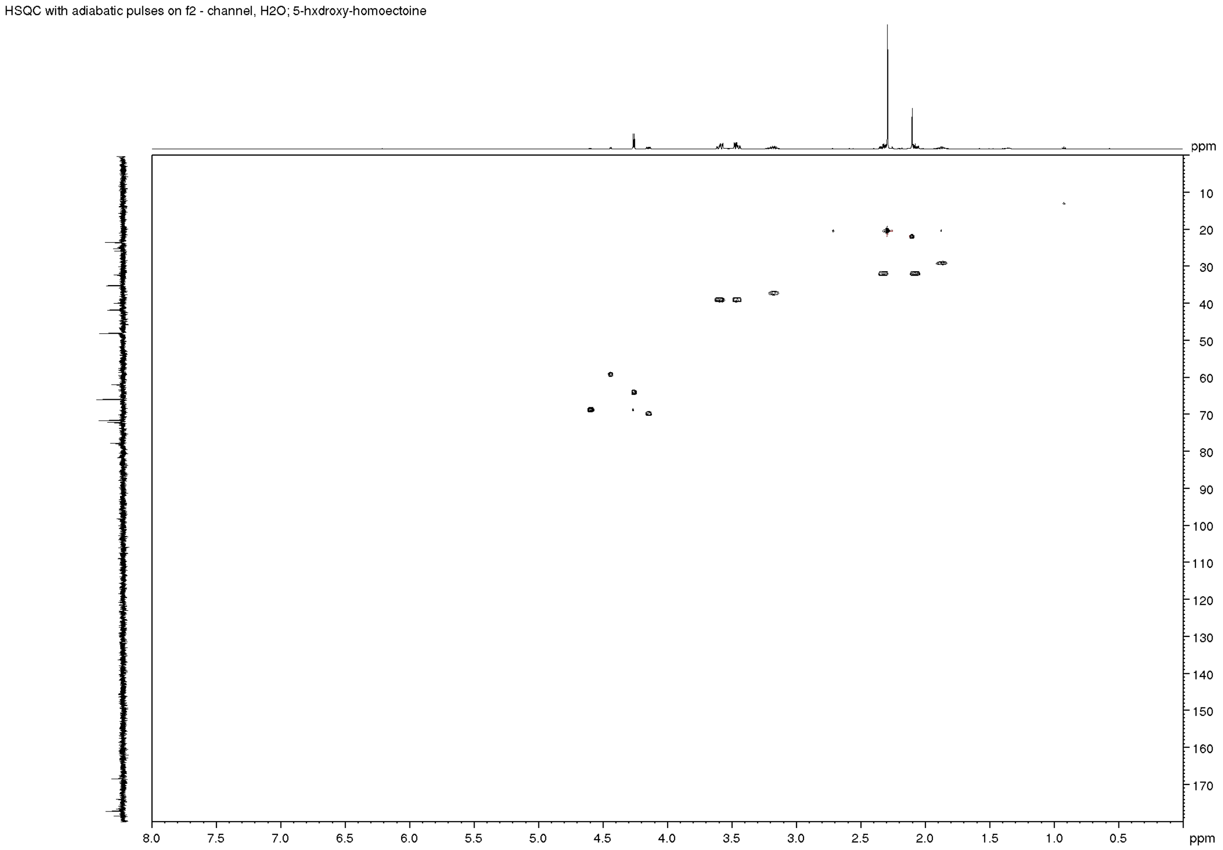

Supplementary Figure 14. HSQC spectrum of 5-hydroxyhomoectoine.

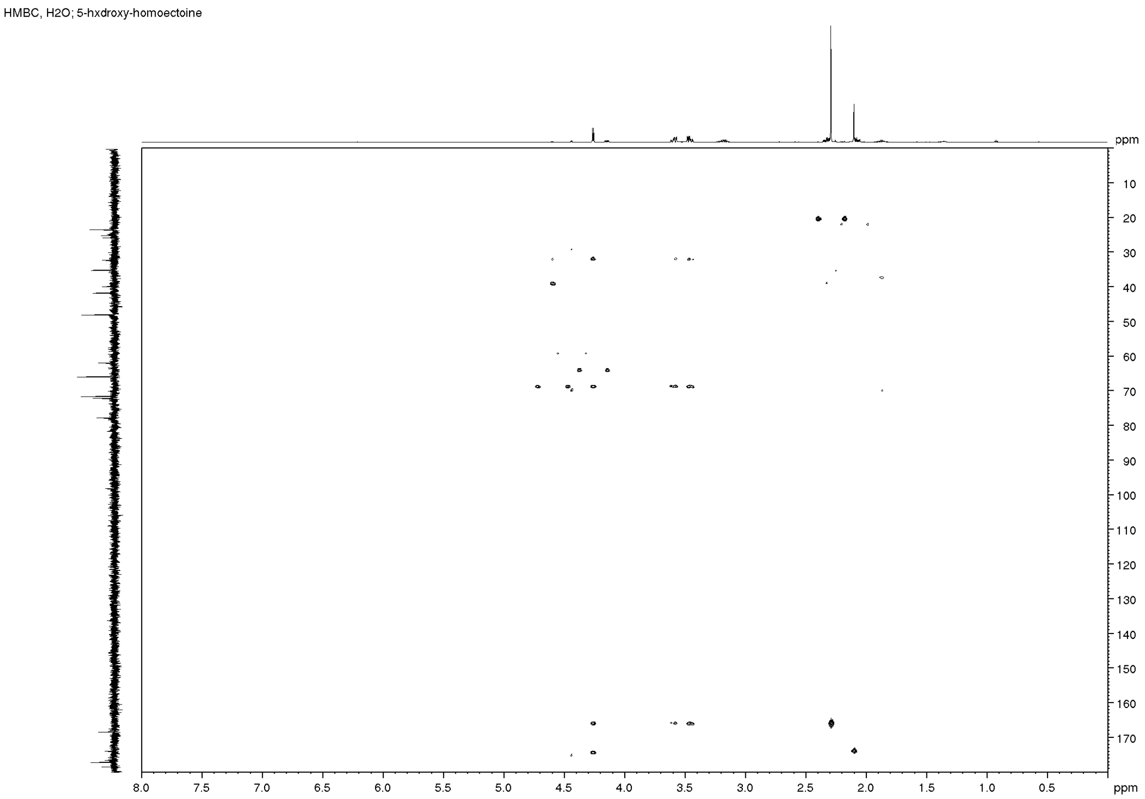

Supplementary Figure 15. HMBC spectrum of 5-hydroxyhomoectoine.

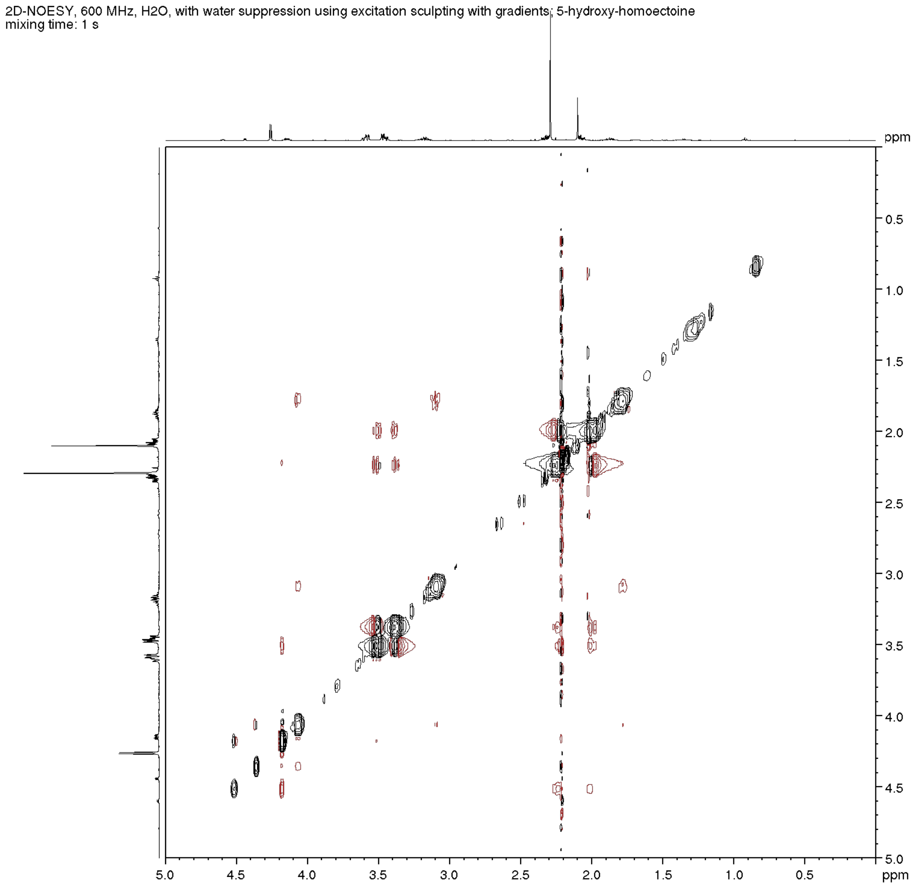

Supplementary Figure 16. NOESY spectrum of 5-hydroxyhomoectoine.

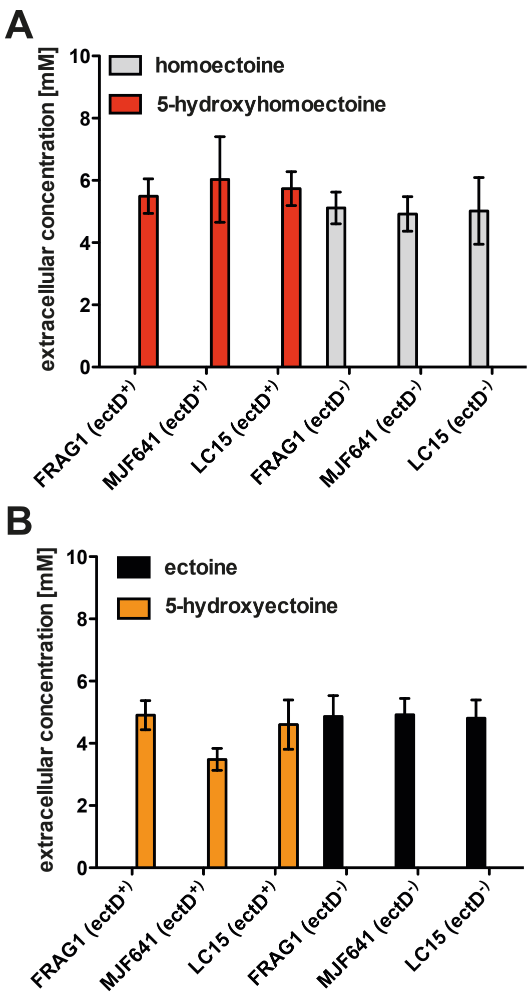

Supplementary Figure 17. Influence of mechanosensitive channels on the release of 5-hydroxyectoine and 5-hydroxyhomoectoine. The *E. coli* strains FRAG1 (wild-type), MJF641 (FRAG1 *mscL mscK mscS mscM*) and LC15 (*otsA1*::Tn*10*), that contained either the empty vector pASK-IBA3 (control), or the *ectD*-expression plasmid pMP41 (*ectD* gene from *P. stutzeri* A1501), were grown in the presence of **(A)** 5 mM homoectoine or **(B)** 5 mM ectoine. The cultures were grown in baffled flasks containing 10 ml of MMA with 0.4 M NaCl. They were incubated for 24 hours after induction of enhanced *ectD* transcription from the TetR-controlled *tet* promoter with the synthetic inducer AHT. Ectoines were quantified in the supernatants using HPCL analysis. The shown data represent the means and standard deviations of at least four independently grown cultures.

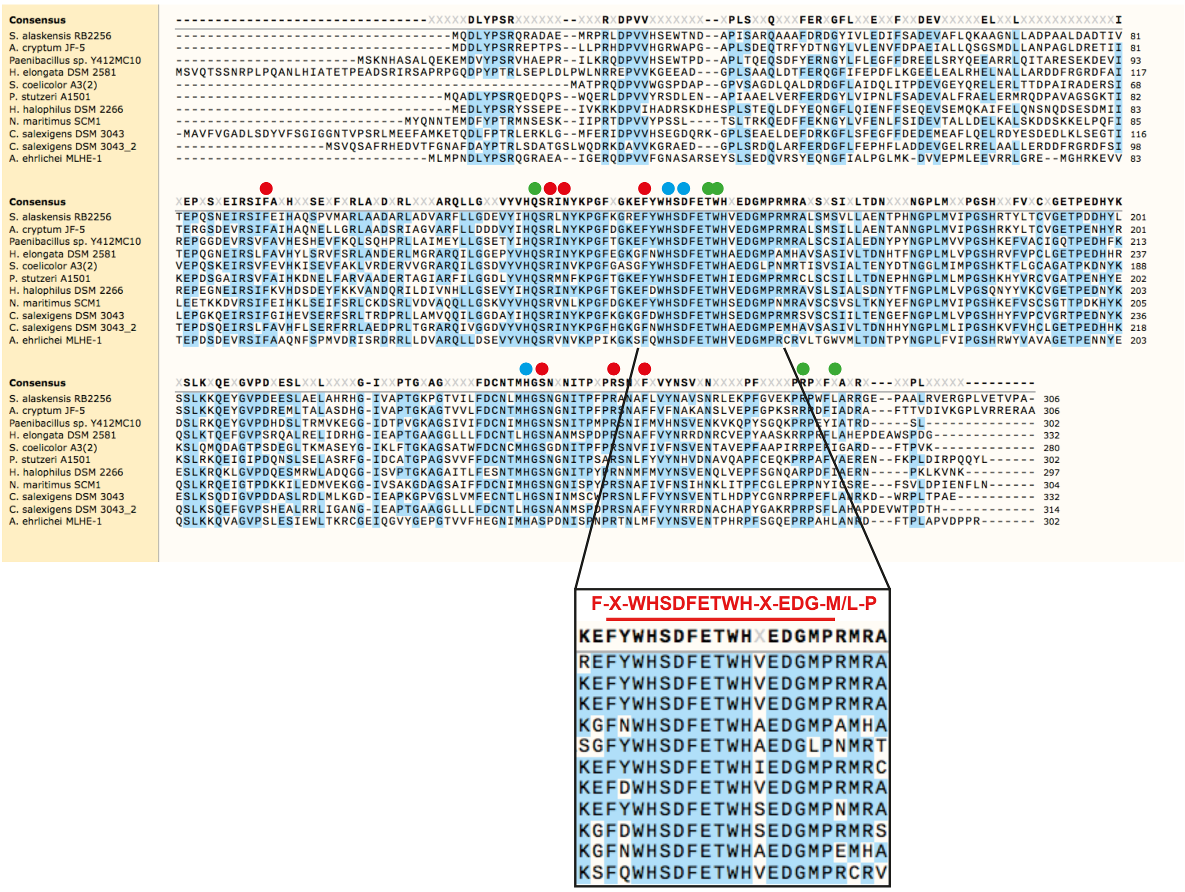

Supplementary Figure 18. Protein sequence alignment of the EctD proteins from *Sphingopyxis alaskensis* (WP_011543221.1), *Acidiphilum cryptum* (WP_012040480.1), *Paenibacillus lautus* (WP_015737572.1), *Halomonas elongata* (WP_013333764.1), *Streptomyces coelicolor* (NP_626134.1), *Pseudomonas stutzeri* (WP_011911424.1), *Halobacillus halophilus* (WP_014643639.1), *Nitrosopumilus maritimus* (WP_012215726.1), *Chromohalobacter salexigens* (WP_011505850.1; WP_011508293.1), *Alkalilimicola ehrlichii* (WP_011628142.1), *Streptomyces chrysomallus* (WP_030590139.1) and *Virgibacillus salexigens* (AAY29689.1) was performed with SnapGene® software (GSL Biotech; snapgene.com). The EctD signature sequence and residues involved in the binding of the reaction product (5-hydroxyectoine) (green), the co-factor 2-oxoglutarate (red) or the iron atom (blue) are highlighted (Reuter et al., 2010; Höppner et al., 2014).

References

Baba, T., Ara, T., Hasegawa, M., Takai, Y., Okumura, Y., Baba, M., et al. (2006). Construction of *Escherichia coli* K-12 in-frame, single-gene knockout mutants: the Keio collection. *Mol Syst Biol* 2**,** 2006 0008. doi: 10.1038/msb4100050.

Blattner, F.R., Plunkett, G., 3rd, Bloch, C.A., Perna, N.T., Burland, V., Riley, M., et al. (1997). The complete genome sequence of *Escherichia coli* K-12. *Science* 277(5331)**,** 1453-1462. doi: 10.1126/science.277.5331.1453.

Edwards, M.D., Black, S., Rasmussen, T., Rasmussen, A., Stokes, N.R., Stephen, T.L., et al. (2012). Characterization of three novel mechanosensitive channel activities in *Escherichia coli*. *Channels (Austin)* 6(4)**,** 272-281. doi: 10.4161/chan.20998.

Epstein, W., and Kim, B.S. (1971). Potassium transport loci in *Escherichia coli* K-12. *J Bacteriol* 108(2)**,** 639-644.

Haardt, M., Kempf, B., Faatz, E., and Bremer, E. (1995). The osmoprotectant proline betaine is a major substrate for the binding-protein-dependent transport system ProU of *Escherichia coli* K-12. *Mol Gen Genet* 246(6)**,** 783-786.

Höppner, A., Widderich, N., Lenders, M., Bremer, E., and Smits, S.H.J. (2014). Crystal structure of the ectoine hydroxylase, a snapshot of the active site. *J Biol Chem* 289(43)**,** 29570-29583. doi: Doi 10.1074/Jbc.M114.576769.

Kuhlmann, A.U., and Bremer, E. (2002). Osmotically regulated synthesis of the compatible solute ectoine in *Bacillus pasteurii* and related *Bacillus* spp. *Appl Environ Microbiol* 68(2)**,** 772-783.

Reuter, K., Pittelkow, M., Bursy, J., Heine, A., Craan, T., and Bremer, E. (2010). Synthesis of 5-hydroxyectoine from ectoine: crystal structure of the non-heme iron(II) and 2-oxoglutarate-dependent dioxygenase EctD. *PloS one* 5(5)**,** e10647. doi: 10.1371/journal.pone.0010647.

Strom, A.R., and Kaasen, I. (1993). Trehalose metabolism in *Escherichia coli*: stress protection and stress regulation of gene expression. *Mol Microbiol* 8(2)**,** 205-210.

Studier, F.W., Rosenberg, A.H., Dunn, J.J., and Dubendorff, J.W. (1990). Use of T7 RNA polymerase to direct expression of cloned genes. *Methods Enzymol* 185**,** 60-89.

Widderich, N., Höppner, A., Pittelkow, M., Heider, J., Smits, S.H., and Bremer, E. (2014). Biochemical properties of ectoine hydroxylases from extremophiles and their wider taxonomic distribution among microorganisms. *PLoS One* 9(4)**,** e93809. doi: 10.1371/journal.pone.0093809.
